# AI-Assisted surgical vision: evaluating YOLOv8 and YOLOv12 for real-time detection in colon cancer surgery

**DOI:** 10.3389/fsurg.2025.1724635

**Published:** 2026-01-13

**Authors:** Li Li, Bin Xuan, Xin Song, Yu Tian, Xiangcai Meng, Jiexia Wen, Tao Zheng, Chenglin Liu, Yimin Wang

**Affiliations:** 1 Department of Surgery, Hebei Medical University, Shijiazhuang, Hebei, China; 2 Department of General Surgery, The First Hospital of Qinhuangdao, Qinhuangdao, China; 3 School of Computer and Communication Engineering, Northeastern University at Qinhuangdao, Qinhuangdao, China; 4 Department of Central Laboratory, First Hospital of Qinhuangdao, Hebei Medical University, Qinhuangdao, China; 5 Department of Imaging, The First Hospital of Qinhuangdao, Qinhuangdao, China; 6 Key Laboratory of Research on Molecular Mechanism of Gastrointestinal Tumors in Qinhuangdao, The First Hospital of Qinhuangdao, Qinhuangdao, China

**Keywords:** artificial intelligence, laparoscopy, colon cancer, surgical treatment, targetdetection task, instance segmentation task

## Abstract

**Objective:**

Current intraoperative navigation systems have shown significant effectiveness for organs with fixed shapes, but they struggle to adapt to the challenges of tissue deformation and displacement in gastrointestinal surgeries. This study evaluates the established YOLOv8 and the emerging YOLOv12 with enhanced feature extraction capabilities, aiming to identify an optimal real-time model for dynamic surgical scenarios to improve procedural efficiency and safety.

**Methods:**

In this multi-center retrospective study, object detection and instance segmentation was achieved by training YOLOv8 and YOLOv12 models on 1,847 images extracted from 22 surgical videos collected across four hospitals nationwide. The models were subsequently validated and tested and performance was rigorously compared using standard metrics, such as precision, recall, mAP@0.5, mAP@0.5–0.95, and the size of the weight file. Furthermore, the clinical applicability of the top-performing models was evaluated via a questionnaire survey.

**Results:**

Both YOLOv8 and YOLOv12 demonstrated competent performance in object detection and instance segmentation tasks. For the test set, YOLOv12 achieved significantly higher recall rates than YOLOv8 in both object detection and instance segmentation (*P* = 0.037 and *P* = 0.031, respectively). Furthermore, when evaluating the YOLOv12 model on the test set, object detection significantly outperformed instance segmentation in terms of mAP@0.5 and recall (*P* = 0.045 and *P* = 0.036, respectively). The weights files of YOLOv8 and YOLOv12 have sizes of 6.8 megabytes (MB) and 6.0 megabytes (MB) respectively. Questionnaire results indicated a trend suggesting that AI-assisted technology has the potential to reduce surgical time and lower the risk of missed lymph node detection among junior surgeons.

**Conclusion:**

In scenarios with limited hardware resources, the object detection task using the YOLOv12 model is strongly recommended to assist in robotic colon cancer surgery, enhancing surgical efficiency and safety.

## 1 Introduction

The rising incidence and mortality rates of colorectal cancer present a significant global public health challenge (1). In China, the ratio of colon to rectal cancer is approximately 1:1 (2). Surgical resection remains a cornerstone of colon cancer treatment (3, 4), encompassing open surgery, traditional laparoscopic surgery, and robot-assisted laparoscopic surgery (RALS), which represents an upgrade of traditional laparoscopic surgery. RALS has advantages such as precise mechanical arm operation, clear three-dimensional vision, and rapid recovery after minimally invasive surgery (5, 6). Despite its benefits, RALS also exhibits prominent disadvantages. In addition to the inherent lack of tactile feedback, a limitation inherited from traditional laparoscopic surgery, RALS requires substantial time for equipment debugging and mechanical arm positioning before the procedure. Consequently, the total operative duration is typically longer than that of traditional laparoscopic surgery. Therefore, a key objective of this project was to enhance surgical efficiency, shorten the total operation time, and thereby improve the prognosis for patients undergoing RALS.

In the field of urology, an AI automatic matching technology, combined with real-time augmented reality technology has been proposed and applied to partial nephrectomy surgeries. The AI-assisted navigation group was confirmed to shorten the operation time and reduce the incidence of complications (7). In the field of neurosurgery, a similar AI approach (i.e., segmentation dictionary learning algorithm) based on magnetic resonance imaging navigation has been applied in brain glioma resection surgeries. Under the condition of ensuring intact neural function and without increasing the risk of infection, the approach allowed the tumor to be removed to the greatest extent (8). In the field of spinal surgery, the combination of AI and augmented reality with robotic systems has achieved gratifying results (9). The successful application of AI in these three fields is based on a common feature: the anatomical positions of the kidneys, brain, and spine are fixed, which is conducive to positioning. However, due to the characteristics of intestinal tissues being prone to deformation and mobility, the above successful cases are difficult to be replicated in gastrointestinal tumor surgeries. Thus, applying the visual AI tool YOLO has become an important method to address gastrointestinal tumor surgeries. Within the domain of gastrointestinal tumor surgery, the use of AI technology has focused on the real-time detection of surgical instruments (10) and the recognition of surgical steps (11). Notably, the use of AI to automatically identify tumor lesions, lymph nodes, and gauze, thereby assisting surgeons in reducing operative time and improving the lymph node clearance rate, remains largely unexplored.

Several methods for the precise preoperative localization of tumor lesions have been extensively explored to date (12–18). Although these approaches have enhanced the rapid and accurate identification of tumor lesions, they often require preoperative marking or the use of specialized and costly equipment or dyes, limiting their widespread applicability. For instance, the more affordable techniques, such as marking with Indian ink or autologous blood (19), are frequently complicated by issues such as local inflammation (20). Furthermore, attempts to use intraoperative digestive endoscopes (21) and other auxiliary methods for locating early lesions have introduced new challenges, such as increasing the need for additional surgical personnel and prolonging the overall operative time. In some cases, endoscope manipulation can cause intestinal dilation, which can compromise the surgical field of vision. Compared with traditional positioning methods, the AI solution proposed in this study does not require any additional equipment or operations. It can be directly integrated into the surgical system, providing a more convenient technical path for clinical application.

The use of YOLO to achieve precise intraoperative navigation represents a significant new direction in research. YOLO is a deep learning-based object detection algorithm operating in real-time. By dividing an image into a grid and directly predicting the category probability, bounding box coordinates, and confidence score for each target grid, it achieves an efficient balance between detection speed and precision, making it widely applicable in diverse scenarios such as real-time monitoring, autonomous driving, and medical image recognition. YOLOv8 and YOLOv12 are successive iterative versions within the YOLO algorithm series. They adhere to the core “single detection” principle of YOLO, while incorporating optimizations and enhancements to the network structure, loss function, detection precision, and speed to meet the requirements of increasingly complex scenarios. The YOLOv8 model has demonstrated extensive application potential across various fields, particularly in medicine. Its application in medical image analysis, disease detection (22–27), surgical navigation (28–30), auxiliary diagnosis (31–38), and the development of prediction models (39) has seen a gradual increase in recent years. Due to its rapid detection speed and high precision, YOLOv8 has become a major focus for researchers. Notably, improvements have been made in its model structure to enable it to perform specific tasks and maximize its performance (40–44). Following the official launch of YOLOv12 in February 2025, extensive performance analyses has been conducted across numerous fields. YOLOv12 has shown excellent performance in the field of medical (45, 46), and in navigation and driving (47, 48), security monitoring (49), industrial automation (50–53), agricultural automation (54–59), and environmental monitoring (60, 61). A comparison of the structural and model performance of YOLOv8 and YOLOv12 is presented in Supplementary Tables S1–S3 and Supplementary Figure S1.

An evaluation of real-time detection tools in robotic surgery confirmed that the convolutional neural network based on the YOLO architecture achieved a real-time detection speed of 48.9 frames per second, with an average precision of 0.722 on the test set. The detection speeds of YOLOv8 and YOLOv12 in this experiment were both greater than 300 frames per second, and the average precision of both was greater than 0.9. Both models have significant advantages when applied in robotic surgery. In the performance experiments of models such as the YOLO family and RT-DETR in the detection of retinal optical coherence tomography lesions: YOLOv12 achieved the best balance between precision and computational efficiency, with an inference time of 4.9 ms on the AROI dataset, mAP@50 of 0.712, which was superior to YOLOv9, YOLOv10, and YOLOv11. YOLOv8 had comparable inference time to YOLOv12. RT-DETR performed significantly worse (45). Additionally, the development of hybrid deep learning architectures (ViT-GRU and GoogleNet-SVM) to achieve precise detection and classification of brain tumors based on magnetic resonance imaging, achieved an accuracy of the ViT-GRU model and GoogleNet-SVM model of 95.35% and 92.60%, respectively (62). This performance is comparable to that of YOLOv8 and YOLOv12. Although the hybrid deep learning architectures performed well in terms of performance, YOLOv8 and YOLOv12 are single-stage object detection models, having the advantage of a simple structure, and can balance detection precision and efficiency. For surgical video detection scenarios with high real-time requirements, YOLOv8 and YOLOv12 are recommended as the first-choice models. This study selects YOLOv8 and YOLOv12 as the core models based on following key considerations. First, real-time in surgical scenarios place extremely high demands on the balance between model inference speed and precision. The unique single-stage detection architecture of the YOLO series enables millisecond-level inference, and its exceptional real-time performance has been validated in a series of visual tasks, making it particularly suitable for the instant analysis needs of surgical video streams. Second, YOLOv8, as a widely validated and mature version, provides a reliable performance benchmark for this research, while YOLOv12 incorporates the latest architectural optimizations, representing the forefront of this technological trajectory. Through a systematic comparison of these two model generations, we ensure the comparability of research conclusions while objectively evaluating the practical improvements in surgical segmentation efficacy driven by technological evolution.

In summary, the main contributions and innovations of this work can be described as follows. First, YOLOv8 and YOLOv12 were applied in robot-assisted radical colon cancer surgery for auxiliary recognition. This confirmed the feasibility and clinical applicability of using tumors, lymph nodes, and gauze as the primary research targets. Both models exhibited superior learning and generalization abilities, providing technical support for enhancing surgical efficiency and reducing intraoperative misjudgments, thus indirectly improving patient prognosis. Second, a systematic comparison of the performance of YOLOv8 and YOLOv12 in both target detection and instance segmentation tasks was conducted within the context of robot-assisted laparoscopic radical colon cancer surgery. This provided a crucial data reference for model selection in this specialized domain. Additionally, with regard to robotic colon surgery, this study was the first to evaluate the utility of YOLOv12. Finally, the integration of AI technology with robotic surgery presents novel ideas for future research and development in intelligent surgical systems.

## 2 Materials and methods

### 2.1 Data collection and frame annotation

Between April and December 2024, a total of 22 complete robot-assisted laparoscopic radical colon cancer surgery videos were collected from four major tertiary hospitals: Peking Union Medical College Hospital (*n* = 11), Qinhuangdao First Hospital (*n* = 5), Harbin Affiliated Hospital (*n* = 4), and Peking University Affiliated Hospital (*n* = 2). The patient cohort consisted of 12 males and 10 females, with a median age of 61 (IQR: 48–74) years and a median body mass index of 21.2 kg/cm^2^ (IQR: 18.3–25.3). For dataset creation, 5–10 min of footage involving lymph node dissection and tumor lesion localization were extracted from each video. Using Adobe Premiere Pro software, the sampling rate was set to 1 frame per second to extract frames from the video, resulting in 19,156 images, with a resolution of 3,840 × 2,160 pixels. In the case where the frame data of Peking Union Medical College Hospital is significantly larger, a stratified sampling method based on the light intensity of the surgical scene was adopted. 60% of the frames were selected for the experiment to balance the frame numbers among different hospitals. Manual curation was used to eliminate blurry and highly repetitive frames. When selecting the frames, different lighting conditions, blood infiltration, and the obstruction caused by tissues and surgical instruments were considered to ensure the representativeness of the data and 1,847 frames were selected for the final dataset and annotated (Supplementary Table S4). For the training set and the test set, 1,679 frames were randomly divided from the three hospitals, namely the First Hospital of Qinhuangdao City, the Affiliated Hospital of Harbin, and the Affiliated Hospital of Peking University, into a training set (1,176 frames) and a validation set (503 frames) in a 7:3 ratio. The test set consisted of 168 frames from the videos of 2 patients at Peking Union Medical College Hospital. The dataset was independently labeled by 2 surgeons with over 5 years of clinical experience in general surgery. In case of disagreement between the two physicians, the third physician, a chief physician with over 10 years of experience, served as the gold standard for arbitration. After arbitration, a 100% consensus was reached. The annotation flowchart can be found in Supplementary Figure S2.

### 2.2 Ethical approval, informed consent, and data anonymization

This study was conducted in full compliance with the “Ethical Review Measures for Biomedical Research Involving Humans” and the principles of the Declaration of Helsinki. The study protocol, including all procedures for data handling and patient informed consent, was reviewed and approved by the Institutional Review Board (IRB) of The First Hospital of Qinhuangdao (Approval Number: 2025K-158-01).

To ensure patient privacy and data confidentiality, a rigorous multi-step anonymization process was implemented. All direct personal identifiers were permanently removed from the video files and metadata. The video content was strictly limited to the intraoperative field, excluding any patient-facing footage. Each de-identified video was assigned a unique study code, with the mapping to original data stored in a separate, encrypted log accessible only to the ethics committee for audit purposes. All data were stored on an encrypted server within a secure hospital network, isolated from public internet access.

Written informed consent was obtained from each participant. The consent form explicitly detailed the research purpose, data usage scope, implemented privacy protection measures, and the data management plan (secure archiving for 3 years post-study followed by permanent destruction), in accordance with regulatory requirements.

### 2.3 Experimental methods

#### 2.3.1 Experimental environment and parameters

To ensure the comparability of the research results, all experiments were conducted on the same Lenovo laptop. The hardware configuration included an NVIDIA GeForce RTX 4070 Laptop Graphics Processing Unit and an Intel Core i7 CPU. Training was conducted using the default hyperparameter configuration. YOLOv8 and YOLOv12 were evaluated under the same training conditions. The experimental parameter settings are shown in Table 1.

**Table 1:** Training parameters of YOLOv8 and YOLOv12.

Parameters	YOLOv8 and YOLOv12
Epochs	300 rounds
Input image size	640*640 pixels
Batch Size	16 frames
Momentum setting	0.9
Learning rate	0.001
Weight decay	0.005

#### 2.3.2 Model training, validation, and testing

In this study, the YOLOv8 and YOLOv12 family models were used. Both models were accessed through the official Ultralytics GitHub repository (cloned from https://github.com/ultralytics/ultralytics). Both models were trained and evaluated using their respective pre-trained weights (yolov8n-seg.pt and yolov12n-seg.pt). These weights are natively supported within the Ultralytics framework and can be either automatically downloaded using the command-line interface or specified manually. To verify the stability of the model, 30 cross-validations were conducted on a total of 1,679 frames from both the training set and the validation set. The cross-validation results are presented in Supplementary Tables S5, S6. Data augmentation strategies such as rotation, flipping, cropping and scaling, brightness/contrast adjustment, and noise injection were adopted for the dataset to enhance the robustness of the model.

#### 2.3.3 Evaluation metrics

Precision, was defined as the proportion of correctly predicted positive instances out of all samples predicted as positive (i.e., positive predictive value). Recall was defined as the proportion of actual positive instances that the model correctly identified as positive (i.e., sensitivity). The F1 Score was calculated as the harmonic mean of precision and recall, providing a single metric that balances both factors. The mean average precision (mAP) is a comprehensive metric derived by averaging the average precision across multiple object categories. It was used to measure the overall localization and classification performance of the model across the entire dataset. The intersection over union (IoU) is an important evaluation metric in computer vision tasks, such as object detection and visual tracking. It was used to measure the similarity between the predicted bounding box and the real bounding box by calculating the ratio of the intersection to the union of the two and intuitively reflects the precision of the target detection algorithm's positioning. Different IoU thresholds can be used to measure mAP to provide more detailed insights into the model's performance. Two common variants are mAP@0.5 and mAP@0.5–0.95. Achieving a high mAP score indicates that the detection model not only accurately identifies and locates objects of different categories but also maintains this precision under different detection strictness conditions. Frames per second (fps) is a measure of computational speed, representing the number of still images (frames) the model can process per second in dynamic visual content. Precision means assisting the surgeon in “correctly cutting” and “cleaning up” the tumor and lymph nodes, as well as “accurately tracking” the gauze in the clinical surgical environment. A high recall rate means no missed detections of lymph nodes, tumors, and gauze. The three indicators mAP@0.5, mAP@0.5–0.95, and F1 value were used to evaluate the model's good reporting and no missing reporting of lymph nodes, tumors, and gauze in the surgical environment from different perspectives. (In Supplementary Table S7).

### 2.4 Questionnaire

A survey was conducted among 107 general surgeons to assess their perceptions regarding the efficacy of AI in assisting with surgical techniques (see the questionnaire in the Supplement).

## 3 Results

### 3.1 Data annotation and distribution

**Figure 1:**
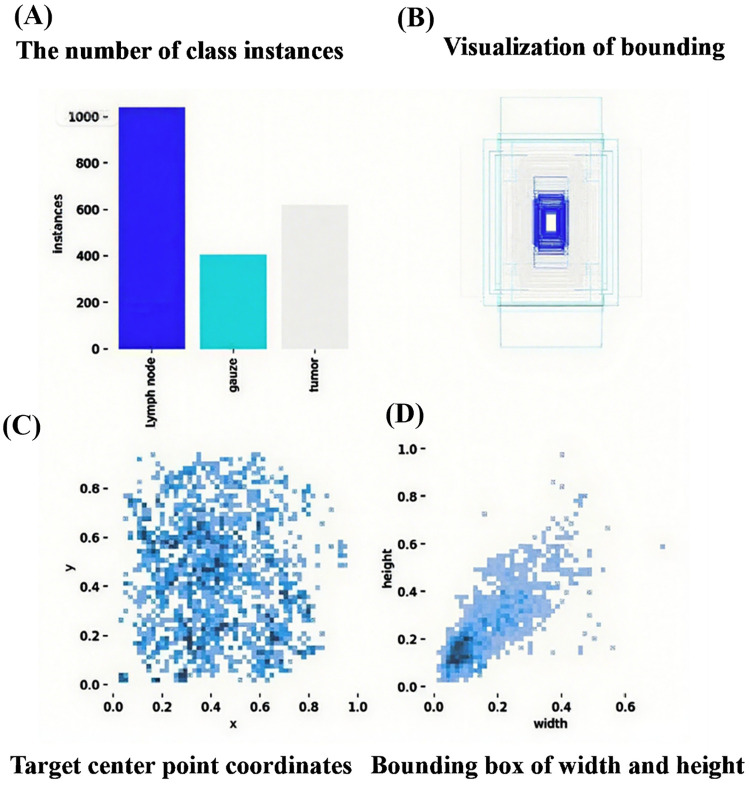
**(A)** The number of instances for each class (lymph node, gauze, tumor); **(B)** Visualization of bounding boxes for target objects; **(C)** Distribution of target center point coordinates (x, y); **(D)** Distribution of bounding box width and height.

The dataset used in this study is a self-constructed dataset comprising three categories: lymph nodes, gauze, and tumors. The consistency index between the two experts, expressed as the Kappa value, was approximately 0.996, indicating a high level of inter-rater reliability. The annotation precision rate was 99.6% and the annotation recall rate was 99.9%. The annotation results were highly consistent with the clinical gold standard, ensuring that the training data can support the clinical practicability of the subsequent model. The details of the expert annotations can be found in Supplementary Table S8, S9. The training dataset was visualized from multiple perspectives, including quantity, category, location, and size. Its characteristics were further analyzed by displaying the spatial distribution patterns and size features of the target bounding boxes (Figure 1 and Supplementary Figure S1). Among the three categories, lymph nodes constituted the largest proportion, whereas gauze represented the smallest, indicating that the data distribution was slightly imbalanced.

### 3.2 Model training and validation results

**Figure 2:**
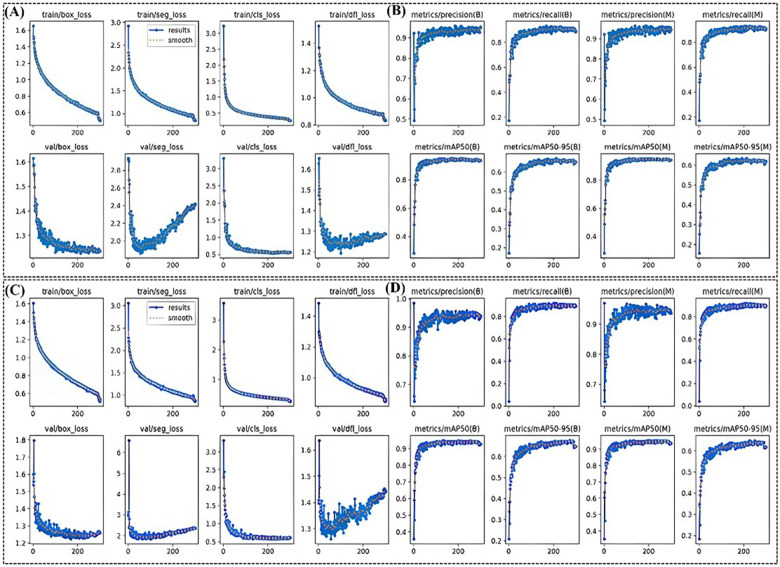
The training results of the **(A,B)** YOLOv8 of and **(C,D)** YOLOv12. In **(A,C)**, the curves decline rapidly before approximately 20 iterations and then gradually level off. In **(B,D)**, the curves increase rapidly before around 20 iterations and then gradually stabilize. In **(A,C)**, the Loss function curves decline rapidly before approximately 20 iterations and then gradually level off. In **(B,D)**, the evaluation indicators curves increase rapidly before around 20 iterations and then gradually stabilize.

The training process for the YOLOv8 and YOLOv12 models was monitored using both loss indicators and evaluation metrics, as depicted in Figure 2. The monitored loss components included bounding box loss, segmentation loss, classification loss, and distribution regression loss. Evaluation metrics included precision, recall, mAP@0.5, and mAP@0.5–0.95. During YOLOv8 training, each loss function exhibits a gradual and steady decrease with increasing iterations, eventually dropping below 1.0 upon convergence. After approximately 20 iterations, the loss functions are observed to decrease rapidly before entering a stable state. This behavior reflects the model's “active learning” phase, indicating that the parameter configuration was well-suited for the current task. Additionally, no substantial differences were observed between the two models at this stage. However, during validation, the loss curves of YOLOv8 were more stable than those of YOLOv12. Both models showed an upward trend in the later stages for distribution regression loss and segmentation loss, suggestive of overfitting. In our study, we proactively addressed potential overfitting through the implementation of several techniques: L2 Regularization: We incorporated L2 regularization (weight decay) with a coefficient (*λ*) of 0.001 during model training. This applies a penalty on the magnitude of the model parameters, discouraging over-reliance on any specific feature and thus reducing model complexity to mitigate overfitting. Data Augmentation: To enhance the diversity and robustness of the training dataset, we applied a series of data augmentation techniques. These included random rotations, horizontal and vertical flips, random cropping and scaling, as well as adjustments to brightness and contrast. Early Stopping: We employed an early stopping strategy during training. This involved continuously monitoring the loss on the validation set. The training process was configured to halt automatically if no improvement in the validation loss was observed for 5 consecutive epochs (patience = 5), thereby preventing the model from over-optimizing to the training data.

YOLOv12 exhibited a more pronounced late-stage increase in distribution regression loss than YOLOv8, whereas the upward trend in the segmentation loss function curve of YOLOv8 was significantly smaller than that of YOLOv12. As shown in Figure 2, the evaluation metric curves (precision, recall, mAP@0.5, and mAP@0.5–0.95) showed rapid increases after approximately 20 iterations, and then stabilized. YOLOv8 showed a steadier improvement than YOLOv12, indicating slightly better learning efficiency and convergence during validation. Table 2 provides a qualitative comparison of the rate of change in the loss functions during training and validation for both models. Overall, the continuous decrease in training and validation losses suggested that both models successfully learned effective features and possess good overall convergence and detection performance.

**Table 2:** Summary table of loss function results of YOLOv8 and YOLOv12.

Class	Train	Valid	Train	Valid	Train	Valid	Train	Valid
	/box_loss	/box_loss	/seg_loss	/seg_loss	/cls_loss	/cls_loss	/dfl_loss	/dfl_loss
Initial value of YOLOv8	1.65622	3.23664	2.9235	2.93479	1.52835	1.61481	3.29966	1.62819
Final value of YOLOv8	0.50285	0.25992	0.84451	2.41573	0.83171	1.23676	1.23676	1.23676
Rate of change of YOLOv8	−69.63%	−91.96%	−71.11%	−17.69%	−45.58%	−23.37%	−62.51%	−24.04%
Initial value of YOLOv12	1.60714	3.57422	3.05925	2.96019	1.48535	1.60106	3.31497	1.61025
Final value of YOLOv12	0.53771	0.26598	0.88309	2.36068	0.86184	1.25943	0.61378	1.44083
Rate of change of YOLOv12	−66.54%	−92.55%	−71.13%	−20.25%	−41.99%	−21.33%	−81.48%	−10.52%

**Figure 3:**
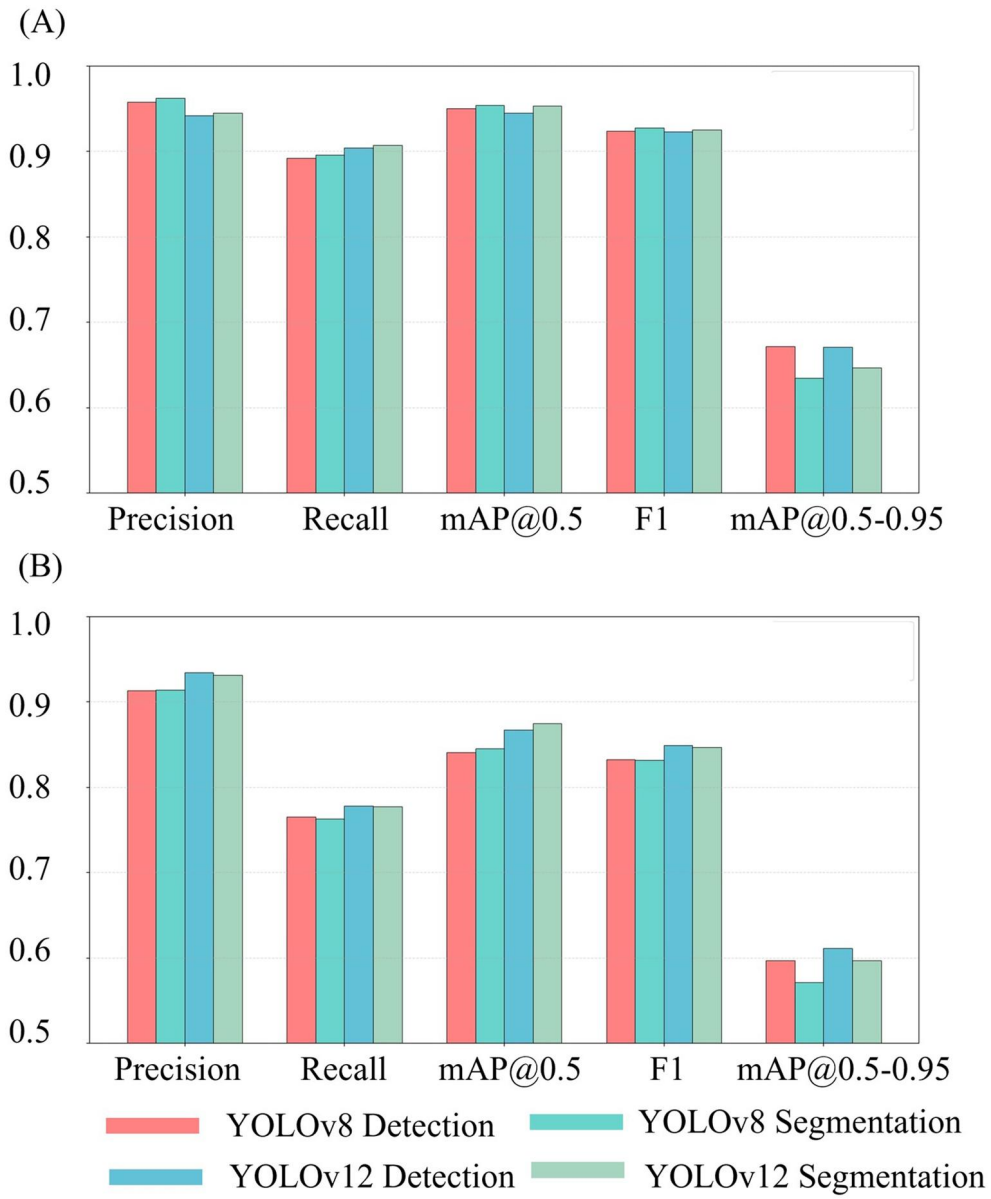
**(A)** Verification results of YOLOv8 and YOLOv12; **(B)** Test results of YOLOv8 and YOLOv12. The bar charts representing each indicator in **(A)** are all taller than those in **(B)** The bar charts representing each indicator in **(A)** are all taller than those in **(B)**.

In the target detection task, the overall average metrics for YOLOv8 vs. YOLOv12 were 0.958 vs. 0.942 for precision, 0.892 vs. 0.904 for the recall rate, 0.95 vs. 0.945 for mAP@0.5, 0.672 vs. 0.671 for mAP@0.5–0.95, and 92.38% vs. 92.26% for the F1 score. For the instance segmentation task, the comparison results were 0.962 vs. 0.945 for precision, 0.896 vs. 0.907 for recall, 0.95 vs. 0.945 for mAP@0.5, 0.635 vs. 0.647 for mAP@0.5–0.95, and 92.26% vs. 92.56% for the F1 score. Overall, both YOLOv8 and YOLOv12 demonstrated excellent performance across both tasks for detecting lymph nodes, gauze, and tumors. Notably, the category-specific precision for both models exceeded 0.9, and the recall rates were all greater than 0.8. Details of the results for both YOLOv8 and YOLOv12 are presented in Table 3, Figure 3. (Supplementary Figure S4, Supplementary Tables S10, S11).

**Table 3:** Comparison of validation experimental results between YOLOv8 and YOLOv12.

Parameters	Type of model	All (LN + Gauze + Tumor) 95% CI		LN	Gauze	Tumor
Precision	YOLOv8	0.958	(0.904, 0.981)	0.969	0.934	0.971
Recall rate		0.892	(0.836, 0.948)	0.882	0.915	0.879
mAP@0.5		0.95	(0.896, 0.986)	0.962	0.966	0.924
mAP@0.5–0.95		0.672	(0.614, 0.728)	0.659	0.802	0.555
F1 score (%)		92.38	(87.32, 96.57)	92.34	92.44	92.27
Precision	YOLOv8-seg	0.962	(0.904, 0.981)	0.967	0.94	0.979
Recall rate		0.896	(0.833, 0.954)	0.879	0.921	0.887
mAP@0.5		0.954	(0.900, 0.982)	0.957	0.967	0.939
mAP@0.5–0.95		0.635	(0.578, 0.689)	0.634	0.724	0.546
F1 score (%)		92.78	(90.10, 95.09)	92.09	93.04	93.07
Precision	YOLOv12	0.942	(0.887, 0.982)	0.957	0.937	0.931
Recall rate		0.904	(0.850, 0.959)	0.901	0.932	0.879
mAP@0.5		0.945	(0.888, 0.981)	0.954	0.956	0.923
mAP@0.5–0.95		0.671	(0.613, 0.727)	0.639	0.796	0.579
F1 score (%)		92.26	(87.91, 94.91)	92.81	93.45	90.42
Precision	YOLOv12-seg	0.945	(0.889, 0.982)	0.957	0.926	0.951
Recall rate		0.907	(0.852, 0.961)	0.901	0.921	0.898
mAP@0.5		0.953	(0.898, 0.980)	0.955	0.956	0.948
mAP@0.5–0.95		0.647	(0.592, 0.701)	0.624	0.739	0.579
F1 score (%)		92.56	(88.50, 96.19)	92.81	91.80	92.37

LN, lymph node; mAP, mean average precision; CI, confidence interval.

**Figure 4:**
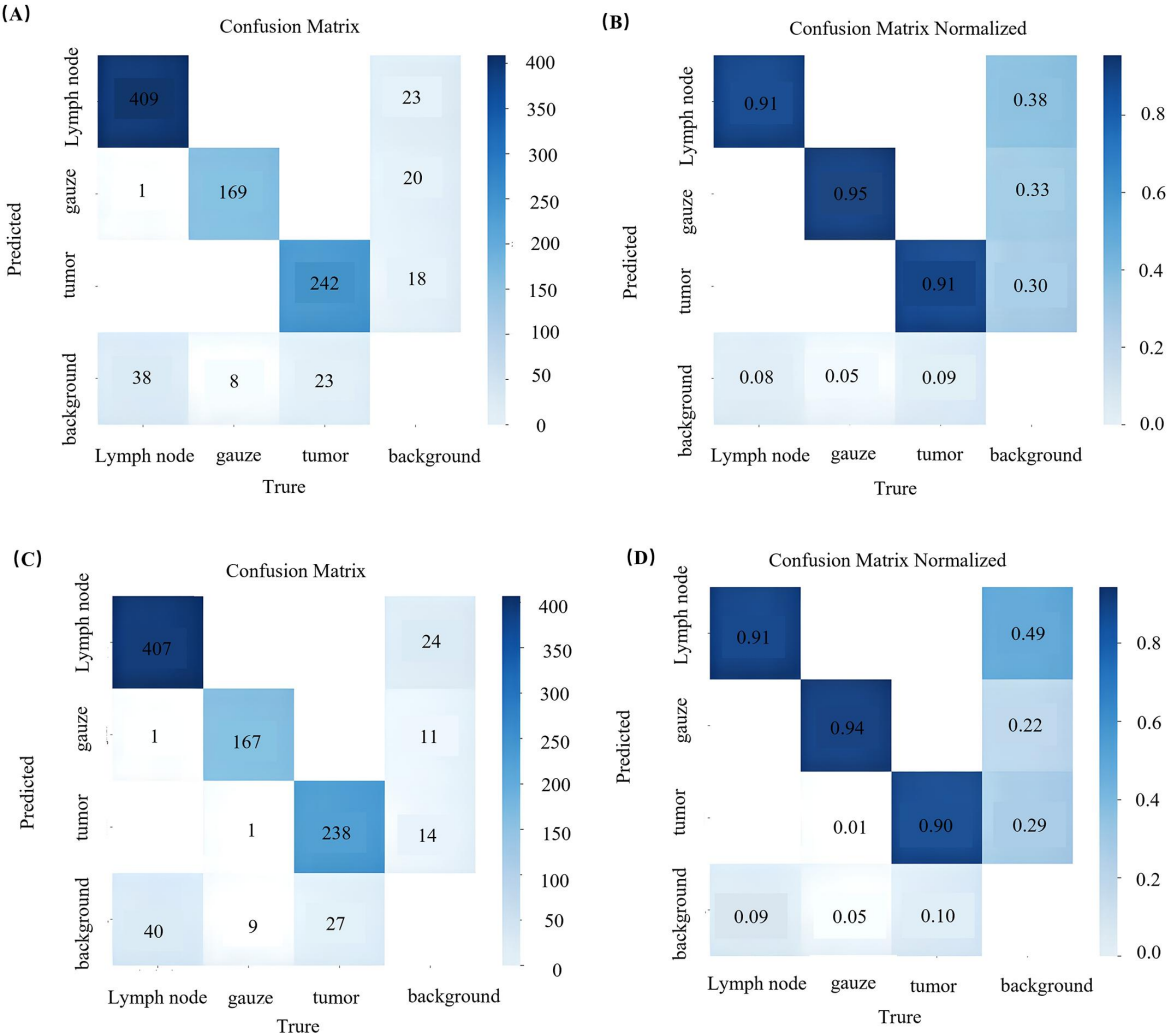
The confusion matrix of experimental results of sample quantity and proportion **(A,B)** of YOLOv8 and **(C,D)** of YOLOv12.

Analysis of the confusion matrix (Figure 4) revealed that the YOLOv8 model had the strongest predictive ability for gauze, correctly identifying 167 cases, accounting for 97% of instances. This was followed by tumors, with 240 correct identifications (91%), and finally lymph nodes, with 401 correct identifications (90%). Misclassifications for YOLOv8 included one case of a lymph node incorrectly predicted as gauze and one case of gauze incorrectly predicted as a tumor. Similarly, the YOLOv12 model showed its strongest performance in predicting gauze, correctly identifying 167 cases (94%), followed by lymph nodes, with 407 correct identifications (91%), and finally tumors, with 238 correct identifications (90%). Misclassifications for YOLOv12 were the same as for YOLOv8, namely, one case of a lymph node incorrectly predicted as gauze and one case of gauze incorrectly predicted as a tumor.

### 3.3 Model test results

In the object detection task, the overall average test metrics for YOLOv8 vs. YOLOv12 were: 0.913 vs. 0.934 for precision, 0.765 vs. 0.778 for recall, 0.841 vs. 0.867 for mAP@0.5, 0.597 vs. 0.661 for mAP@0.5–95, and 83.25% vs. 84.89% for the F1 score. For the instance segmentation task, the corresponding comparison results were 0.914 vs. 0.931, 0.763 vs. 0.777, 0.845 vs. 0.875, 0.571 vs. 0.597, and 83.17% vs. 84.70%. All indicators showed a downward trend when compared with their respective validation set, which suggests a slightly poorer generalization ability in both models. Despite this drop, the precision remained above 0.8 and the recall rate remained above 0.7, indicating that both models had acceptable overall performance in detecting lymph nodes, gauze, and tumors. A comparison of task performance on the test set revealed internal model strengths. Details of the results for both YOLOv8 and YOLOv12 are presented in Figure 4, Table 4. (Supplementary Figure S5, Supplementary Tables S10, S11).

**Table 4:** Comparison of testing experimental results between YOLOv8 and YOLOv12.

Parameters	Type of model	All (LN + Gauze + Tumor) 95% CI		LN	Gauze	Tumor
Precision	YOLOv8	0.913	(0.901, 0.982)	0.934	0.865	0.941
Recall rate		0.765	(0.826, 0.977)	0.725	0.798	0.77
mAP@0.5		0.841	(0.881, 0.988)	0.835	0.856	0.831
mAP@0.5–0.95		0.597	(0.624, 0.731)	0.579	0.755	0.457
F1 score (%)		83.25	(87.10, 96.46)	81.63	83.01	84.69
Precision	YOLOv8-seg	0.914	(0.903, 0.985)	0.934	0.864	0.942
Recall rate		0.763	(0.832, 0.952)	0.725	0.795	0.77
mAP@0.5		0.845	(0.870, 0.980)	0.835	0.856	0.842
mAP@0.5–0.95		0.571	[0.571, 0.688]	0.557	0.679	0.478
F1 score (%)		83.17	[89.11, 94.94]	81.63	79.05	84.73
Precision	YOLOv12	0.934	(0.886, 0.978)	0.944	0.921	0.936
Recall rate		0.778	(0.847, 0.963)	0.737	0.837	0.759
mAP@0.5		0.867	(0.875, 0.974)	0.828	0.907	0.867
mAP@0.5–0.95		0.611	(0.610, 0.725)	0.543	0.775	0.513
F1 score		84.89	(87.90, 94.92)	82.77	87.70	83.82
Precision	YOLOv12-seg	0.931	(0.881, 0.980)	0.944	0.903	0.945
Recall rate		0.777	(0.848, 0.956)	0.738	0.821	0.77
mAP@0.5		0.875	(0.872, 0.975)	0.828	0.906	0.89
mAP@0.5–0.95		0.597	(0.586, 0.703)	0.533	0.725	0.533
F1 score		84.70	(88.45, 96.12)	82.84	86.00	84.85

LN, lymph node; mAP, mean average precision; CI, confidence interval.

### 3.4 Training efficiency and adaptability

An analysis of training efficiency was conducted by examining model parameters and computational costs. YOLOv12 possessed 2,761,345 parameters and had a computational cost of 9.7 GFLOPs, compared with 3,258,649 parameters and 12.0 GFLOPs for YOLOv8. Correspondingly, the final trained file size for YOLOv12 (6.0 MB) was 0.8 MB smaller than that of YOLOv8 (6.8 MB), suggesting that YOLOv12 was more lightweight and thus easier to deploy in practical applications. Despite its advantage in reducing parameter quantity and computational cost, the training efficiency of YOLOv12 was slightly inferior to that of YOLOv8. Specifically, the training time for YOLOv12 (135 min) was longer than that of YOLOv8 (98 min), whereas the frame rate (333 fps) of YOLOv12 was lower than that of YOLOv8 (370 fps). Nonetheless, both models achieved a frame rate greater than 300 fps, which is sufficient to meet the requirements of high-precision scenarios (Supplementary Table S12). The real-time inference capability of YOLOv12 was further demonstrated through its application to a 3-minute microscopic video (see Supplementary Video 1). Supplementary Figures S5, S6 show the cases where the model successfully and failure identified this video.

### 3.5 Questionnaire survey results

**Figure 5:**
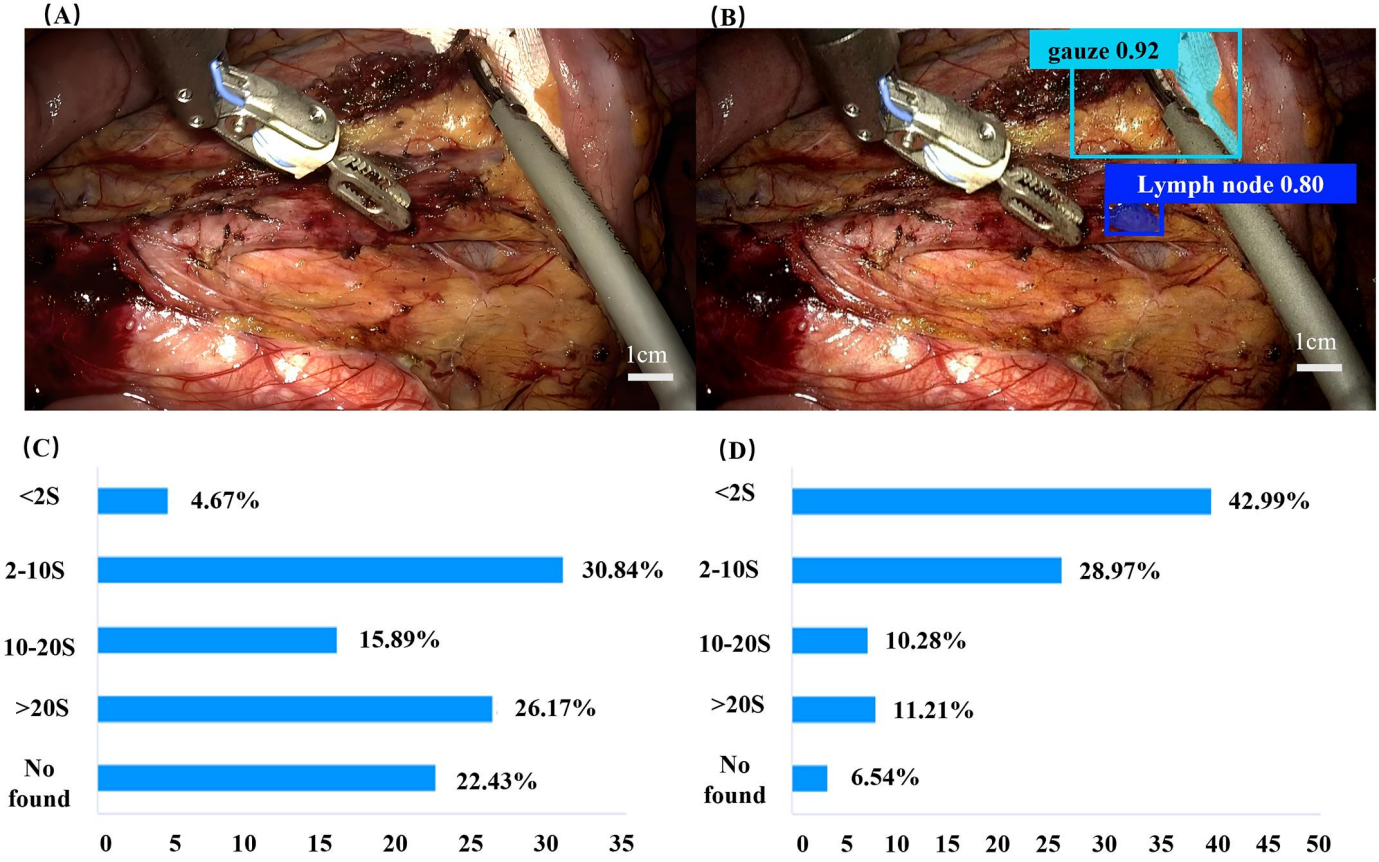
**(A)** The intraoperative images under the robotic-assisted laparoscopic field **(A)** not marked and **(B)** marked by artificial intelligence assistance. **(C)** The participants in the questionnaire provided the distribution of time required for identifying the lymph nodes in **(A,D)** in Figure **(B)**.

As shown in Figure 5, the proportion of surgeons who identified lymph nodes within 2 s rose significantly from 4.67% (without AI) to 42.99% (with AI), an increase of 38.32%. This demonstrated that AI assistance improves lymph node identification efficiency of surgeons. Meanwhile, the proportion of surgeons who failed to identify lymph nodes decreased from 22.43% (without AI) to 22.43% (with AI), a reduction of 15.89%. From the data on the proportion of each age group, the proportion of doctors aged 18–40 who failed to identify lymph nodes without AI assistance was 51.53%, whereas the proportion decreased to 14.37% when AI assistance was available. For doctors aged 50 and above, the proportion of those who failed to identify lymph nodes was 0% regardless of whether AI assistance was available or not. The statistical results of the questionnaire are detailed in Supplementary Tables S13, S15 and Supplementary Figures S7, S8.

## 4 Discussion

Colorectal cancer has an incidence rate of 9.6% and a mortality rate of 9.3%, ranking third and second among all cancers, respectively (1). This condition poses a considerable health burden on the population of China. Surgery remains a primary treatment modality for colon cancer, and robotic-assisted procedures have been increasingly adopted due to their advantages, including reduced bleeding, minimal trauma, and faster postoperative recovery (5, 6). However, the relatively long operation time and absence of tactile feedback highlight the need for innovative approaches to further enhance robotic-assisted surgery. In this study, advanced visual AI technology was employed to upgrade surgical equipment with the goal of assisting surgeons in rapidly identifying tumors, gauzes, and lymph nodes. This approach aims to shorten operation time, improve surgical efficiency, support rapid intraoperative decision-making, reduce missed lymph node detections, and ultimately improve patient prognosis.

The core significance of a high recall rate is to minimize missed detections, which is more crucial in surgical scenarios, than high precision. The shortcomings of precision can be eliminated through manual review by doctors, but the harm caused by missed detections is often irreversible. This aligns with the clinical requirement of “prioritizing safety and having a low tolerance rate” in surgical scenarios. The high recall rate detection of gauze to prevent the presence of foreign objects is a zero-tolerance medical accident prevention measure. High recall rate detection of tumors means avoiding incomplete resection and missed detection of tumors. Ensuring that the surgery is “cleanly removed” is the prerequisite for radical surgery. High recall rate detection of lymph nodes ensures the precision of cancer staging and treatment plans. Missed detection of metastatic lymph nodes can lead to an underestimated stage, thereby affecting optimal selection of treatment plans and increasing the risk of recurrence. Therefore, the high recall rate indicator, as the most core focus of the model, directly relates to the postoperative quality of life and long-term prognosis of patients. mAP@0.5, mAP@0.5–0.95, and F1 scores are comprehensive evaluations of recall rate and precision in different dimensions.

**Figure 6:**
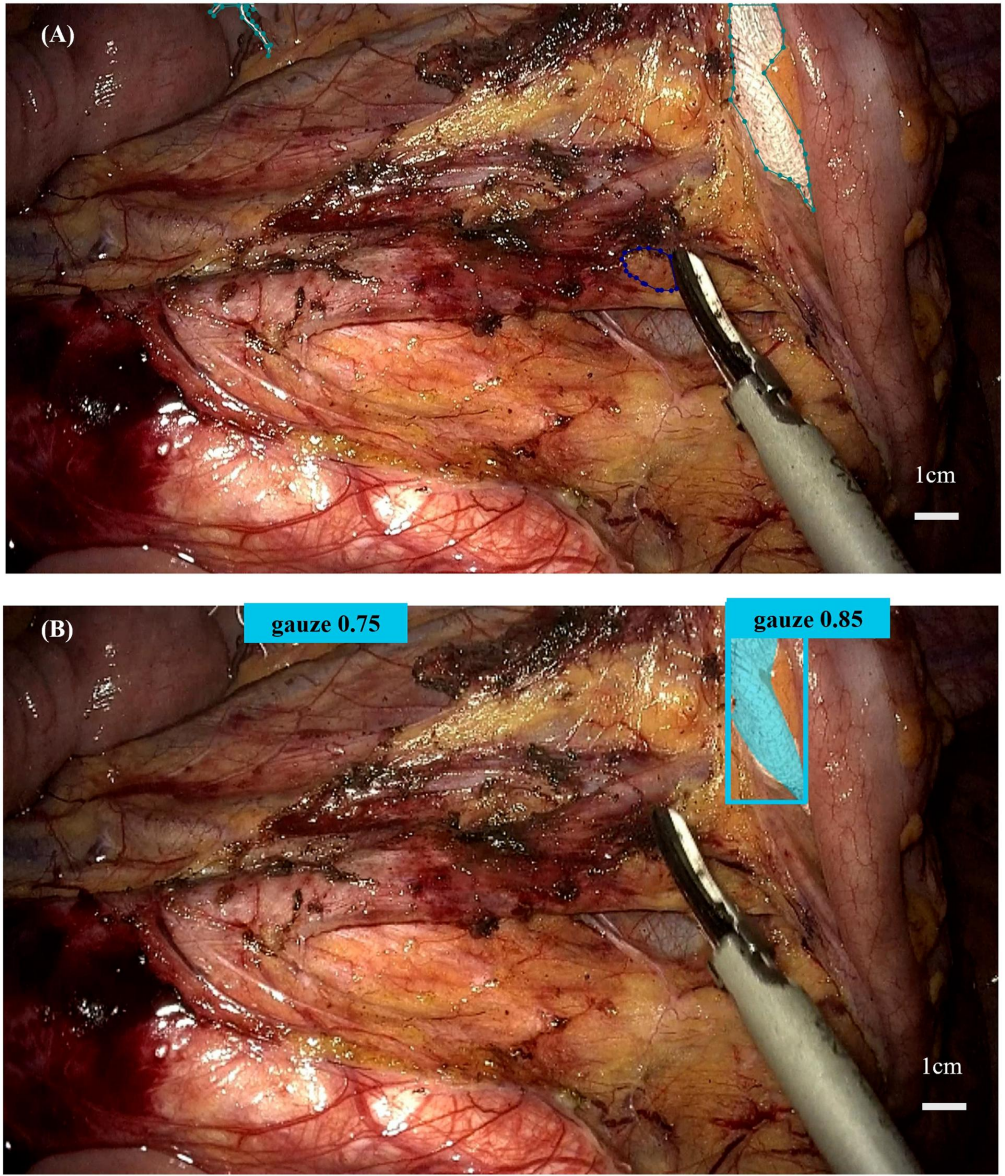
**(A)** The annotation status of the target detection object; **(B)** The successful and unsuccessful performances in video detection.

The morphology of lymph nodes varies considerably, making them difficult to identify in complex surgical environments. Gauze, a commonly used item during surgery, can also present challenges, particularly when stained with blood, as surgeons may spend additional time locating it. Furthermore, the colon is susceptible to deformation and lacks structural stability, which can prolong lesion localization and, in severe cases, result in medical errors such as missed or incorrect resections. To address these three key factors that impede surgical efficiency, we applied, for the first time, multi-center video data based on the latest deep learning frameworks using YOLOv12, the most recent member of the YOLO family, and the widely adopted YOLOv8. Our findings demonstrate that both YOLOv8 and YOLOv12 achieve high detection and recognition precision. Verification results across different categories show precision values exceeding 0.9 and recall rates above 0.8, consistent with previous studies (35, 36, 53, 55, 56, 63, 64). In testing, both models maintained precision and recall rate above 0.8 and 0.7, respectively. While test result was marginally lower than validation metrics, YOLOv8 and YOLOv12 models consistently identified surgical targets in real-time, demonstrating potential to shorten operation time, facilitate rapid intraoperative decision-making, and reduce missed detections. Moreover, inference applications of the model conducted in real-time surgical videos suggest that the model was suitable for deployment for real-time detection and navigation systems during surgical procedures. In Figure 6 are shown two pieces of gauze with partial occlusion, severe occlusion and a small amount of blood staining. Both pieces of gauze were successfully detected, with confidence levels of 0.75 and 0.85 respectively; however, the lymph node failed to be detected due to the obstruction by surgical instruments. But in Figure 5(B), the same lymph node was successfully identified without surgical obstruction, and the confidence level was as high as 0.80. A possible reason for the failure of lymph node detection is that when a small target is obstructed by surgical instruments, the feature extraction dimension is insufficient. To improve measures for failed cases, additional frames should be supplemented that show the lymph nodes being obstructed by tissues or surgical instruments. This will improve the model's robustness and to introduce an attention mechanism to focus on the local area.

The research results of this experiment indicate that: in terms of the recall rate on the test set, YOLOv12 outperforms YOLOv8 in both object detection and instance segmentation tasks (*P* < 0.05). Additionally, when evaluating the YOLOv12 model on the test set, object detection significantly outperforms instance segmentation in terms of mAP@0.5 and recall rate (*P* < 0.05).The stronger generalization ability of YOLOv12 relative to YOLOv8 was likely attributable to the structural optimization of the former. Although the framework of YOLOv12 retains the fundamental architecture of the YOLO family, it differs from YOLOv8 through its attention-centered design. This modification enhances precision and efficiency while maintaining real-time detection capability. The YOLOv12 model is trained using a composite loss function, comprising box loss (weight = 7.5), class loss (weight = 0.5), and distribution focal loss (weight = 1.5) (61). The integration of these three elements aims to optimize the performance of the object detection system. Meeting clinical needs necessitates achieving a high recall rate to ensure the accurate identification of more lymph nodes and a lower rate of missed detections, making this metric critically important (65). Across all categories, the recall rates for YOLOv12 during both training and verification sets, were superior to those of YOLOv8. Although YOLOv8 demonstrated a faster frame rate and shorter training duration, a comparison of efficiency and adaptability showed it demanded higher computational power, a greater number of parameters, and a larger file size. In contrast, YOLOv12 satisfied real-time requirements, exhibited better generalization during testing, and achieved relatively higher recall rates. Consequently, for the detection of lymph nodes, spongy tissues, and tumors, YOLOv12 is recommended for actual deployment under limited hardware resources.

Statistical analysis of the questionnaire data shows that when surgeons are assisted by artificial intelligence (AI), the time taken to identify lymph nodes is significantly reduced (*P* < 0.001). There is no statistically significant difference in the cases of unidentifiable lymph nodes among doctors of different ages, regardless of whether AI assistance is provided. The possible reason for this non-significant statistical result is the insufficient sample size. In the future, the scale of the questionnaire survey can be expanded for further verification. However, for junior surgeons, there is a tendency for the number of unrecognized lymph nodes to decrease when assisted by AI. Based on the above results, when AI assistance is available, the time for lymph node resection is significantly shortened, and junior surgeons are more likely to rely on AI assistance. This indicates that AI—assisted technology is expected to improve surgical efficiency, make up for the lack of experience of junior surgeons, and reduce the missed—detection rate of lymph nodes. The results indicate that applying this technology reduces the risk of missed lymph node detection during surgery and enhances the efficiency of lymph node identification, potentially influencing both patient prognosis and quality of life (65–67). The National Quality Forum in the United States recognizes the retrieval of at least 12 lymph nodes as a surgical quality indicator for colorectal cancer. Furthermore, lymph node retrieval rates are closely associated with postoperative outcomes in colorectal cancer (65). A higher retrieval rate (typically ≥12) correlates with improved survival, lower recurrence, and more accurate pathological staging (66–68).

This study only involved 22 surgical videos, resulting in a small sample size. The limited availability of robotic colon cancer surgeries in China at an early stage also contributed to the insufficient sample size. The small sample size may lead to poor generalization ability and overfitting of the model. With the development of robotic radical colon cancer surgery, future research should expand the sample size by collecting more patient videos to enhance the applicability of the model. Secondly, this study did not undergo external validation, raising doubts about the generalization ability of the model on external data. The videos used in this experiment were all 3D laparoscopic surgery videos from the Condo robot. For many hospitals that only have 2D laparoscopic surgery capabilities, the applicability of the model may be limited. Two-dimensional laparoscopic colon cancer surgery videos should be collected to verify the extraction effect of the model's target detection features in low-resolution surgical scenarios. Additionally, this experimental model was trained only on robot colon cancer surgical frames. It did not cover other tumor types. For the detection of sponges and lymph nodes in other surgical scenarios, the model's performance may be superior to that for tumor detection. The main reason is that the morphological characteristics of different cancers are significantly different. If this model is directly applied to other tumors, the precision may significantly decrease. We can use transfer learning strategies, based on the current model as the pre-trained weights, to gradually build a multi-tumor detection framework.

In summary, this study demonstrates that integrating RALS with AI enhances surgical efficiency, reduces operative time, and improves surgical safety. Further development of this technology is expected to enhance the overall treatment effectiveness and quality of life for colon cancer patients. Subsequent to regulatory approval and clinical deployment, real-world clinical data will be essential to further verify its efficiency and actual impact on patients.

## 5 Conclusion

YOLOv12 and YOLOv8 performed exceptionally well in detecting lymph nodes, gauze, and tumors within the surgical field of robot-assisted laparoscopic radical colon cancer surgery. This study marks the first application of YOLOv12 in this specific surgical domain, successfully verifying its excellent performance, even in lightweight model configurations. The findings confirm the viability of AI-assisted technology for reducing operation time, improving the lymph node clearance rate, and enhancing surgical safety. Ultimately, the results of this project offer novel directions for the future upgrading of robotic surgical equipment.

## Data Availability

The original contributions presented in the study are included in the article/supplementary material, further inquiries can be directed to the corresponding author.
