# Supplementary material for: AI-Assisted surgical vision: evaluating YOLOv8 and YOLOv12 for real-time detection in colon cancer surgery
Source: Front Surg. 2026 Jan 13;12:1724635. doi: 10.3389/fsurg.2025.1724635 (PMC12834723; doi:10.3389/fsurg.2025.1724635)
Supplement: Supplementary file 1 [file Supplementaryfile1.docx]

**1.Introduction**

**Table 1 The research status of YOLOv8**

| Type | Dateset | images | Evaluation indicators | References |
| --- | --- | --- | --- | --- |
| YOLOv8l | USOVA3D 2D fr1 80 20; USOVA3D 2D fr2 80 20 | 1,469 | Precision=97.22%,Recall=75.48%;Precision=99.342%,Recall=81.27% | （23） |
| YOLO  V8X | The Cancer Imaging Archive of the National Cancer Institute of US | 343 | Precision= 85.78%，Recall= 79.40%，mAP@0.5=91.42% | （24） |
| YOLOv8n  ; YOLOv8x | Retrospectively included 209 X-ray images of patients from 2013-2023 | 209 | Recall=91.1%,Precision=81.2%，mAP@50=92.3%;Recall=95.2%, Precision=91% ,mAP@50=95.1% | （25） |
| YOLOv8x;YOLOv8-seg | 11 human cancer cell lines | 14,000 | precision=81.9%96.6%,Recall=96.6%-98.8%,F1=92.7%-97.5% | （26） |
| YOLOv8 | image extraction from TAPP videos | 1098 | F1 = 82%, mAP@0.5 = 87.3% | （28） |
| YOLOv8 | The data set contained 59 brain tumor specimens from 43 patients | 298 | F1 score = 0.61%-0.69% | （31） |
| YOLOv8 | ACNE04; Generated using StyleGAN2 | 4,000 | mAP@0.5:0.95=73.6%,Precision=80.2%,Recall=65.3% | （32） |
| YOLOv8n | MESSIDOR-2 dataset | 1,748 | Accuracy=73.6% | （33） |
| YOLOv8l | Curated Breast Imaging Subset of Digital Database | 1,514 | Precision= 78.81%,Recall  =57.30%，mAP@0.5= 66.80% | （34） |
| YOLOv8l | RSNA 2022 Cervical Spine Fracture Detection Challenge | 9,170 | Precision=90.0%, Recall=89.0%, mAP@0.5=93.5% | （35） |
| YOLOv8s-seg | Nasal endoscopy video screenshots from 52 patients with CRSwNP | 342 | Precision=91%,Recall=83.9%，mAP@0.5= 94.9% | （36） |
| YOLOv8 | Patients with intestinal obstruction who were admitted to the Medical School of Kocaeli University | 700 | Precision= 81.7%，Recall= 75.1%，mAP@0.5= 83.1%，mAP@0.5-95= 45.9% | （39） |

**Table 2 The research status of YOLOv12**

| Type | Dateset | images | Evaluation indicators | References |
| --- | --- | --- | --- | --- |
| YOLOv12 | AROI dataset；OCT5k dataset | 1,136;566 | mAP@0.5=71.2%,mAP@0.5:0.95=48.5%;mAP@50=30.1%,mAP@0.5：0.95=11.1% | （45） |
| YOLOv12n | WCEBleedGen dataset | 2,618 | mAP@0.5=67% | （46） |
| YOLOv12 | RTTS dataset；Foggy Cityscapes | 4,322;5,000 | mAP@0.5=48.2%;mAP@0.5=22.9% | （47） |
| YOLOv12-X | BDD100K dataset; City-Night3K dataset | 18,653;3,560 | mAP@0.5=80.3%,mAP@0.5:0.95=56.8%;mAP@0.5=80.3%,mAP@0.5:0.95=58.4% | （48） |
| YOLOv12 | MOT17 dataset; | 11,235 | Precision=90.9%,recall=77.5%,mAP@0.5=88%,mAP@0.5:0.95=69.5% | （49） |
| YOLOv12n | open-source datasets, social media platforms, and environmental agency custom video recordings | 7,980 | Precision=73%，mAP@0.5=78%，F1=75% | （50） |
| YOLOv12 | images collected from various Internet sources | 3,134 | Precision=84.60%,recall=75.97%,F1=80.10%,mAP@0.5=86.21%, | （51） |
| YOLOv12n | HGI30 dataset | 18,178 | mAP@0.5=90.3%,FPS=93 | （52） |
| YOLOv12n | DsLMF+ dataset | 117,887 | Precision=79.4%-99.4%,recall=73.6%-99.5% | （53） |
| YOLOv12n | lucky bamboo were collected using USB industrial camera | 2,000 | mAP@50=98%,FPS=39.54,reference time=25.3ms, Precision= | (54) |
| YOLOv12x | Tomato-Village dataset | 14,638 | Precision=94.70%,recall=87.30%,mAP@0.5=93.3%,mAP@0.5:0.95=78.3% | （55） |
| YOLOv12m | With eight HIKVISION DS2CD2T46G2-2I RGB | 91,694 | Precision=92.5%,recall=90.8%, mAP@0.5=94.7% | （56） |
| YOLOv12 | Using a Basler acA2040-55uc industrial USB 3.0 | 10,000 | Precision=97%,recall=72.5%,mAP@0.5=83.54%[,F1=97%](mailto:mAP@0.5:0.95=70.25%25,F1=80.47%25)，FPS=45 | （57） |
| YOLOv12 | Weeds Detection dataset | 3,982 | Precision=85%,recall=79.7%,mAP@0.5=87%,mAP@0.5:0.95=58% | （58） |
|  |  |  |  |  |
| YOLOv12s | Means of transportation on Romanian roads | 744 | Precision=99.6%,recall =96.1%, mAP@0.5=98%, | （59） |
| YOLOv12n | IRSTDv1 | 427 | 87.3%,recall=83.3%,mAP@0.5=83.9%,mAP@0.5:0.95=34.5% | （60） |
| YOLOv12x | Garbage in the Ocean Dataset | 5,130 | Precision=87.53%,recall=74.46%,mAP@0.5=83.54%,mAP@0.5:0.95=70.25%,F1=80.47% | （61） |

**Table 3 Comparison between** **YOLOv8 and YOLOv12**

| Model Type | YOLOv8 | YOLOv12 |
| --- | --- | --- |
| Network Structure | 85 floors | 403floors |
| Parameter Quantity | 3,258,649 parameters | 2,761,345 parameters |
| Computational Capacity | 12.0 GFLOPs | 9.7 GFLOPs |


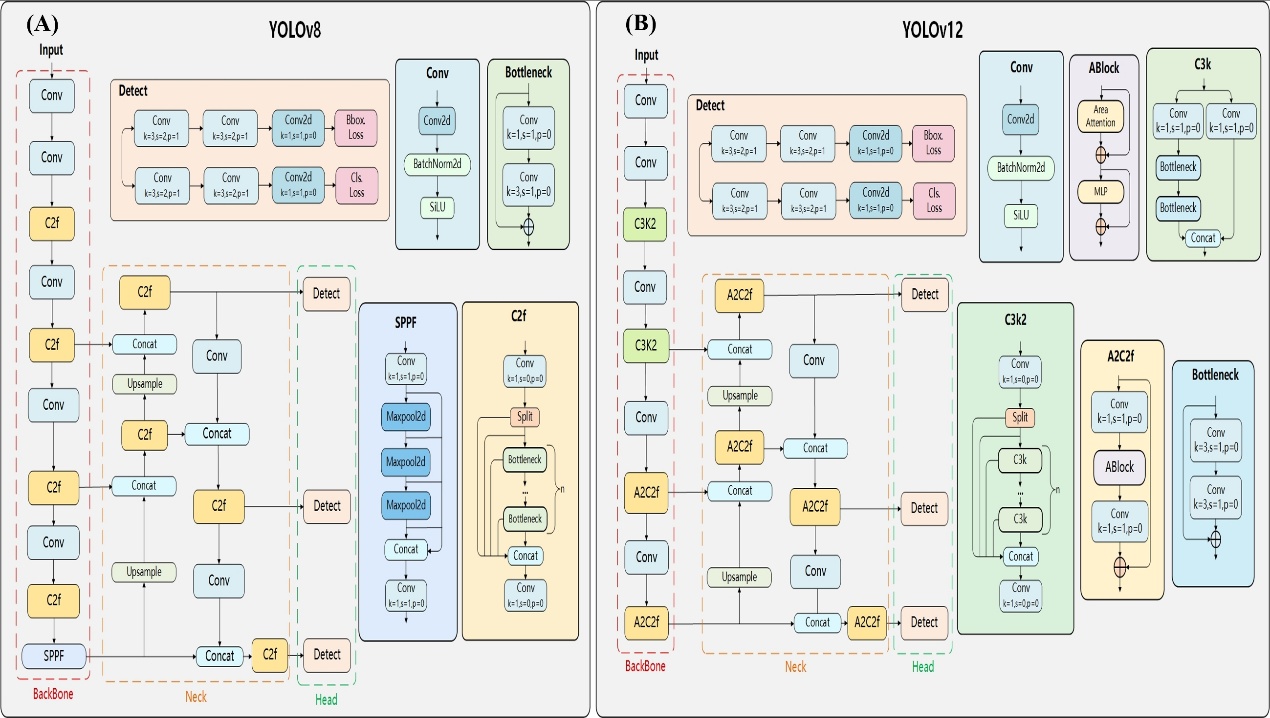


**Figure 1 Overall network architecture of YOLOv8 and YOLOv12**

**Note：A: Network architecture of YOLOv8; B: Network architecture of YOLOv12**

**2.** **Materials and methods
2.1 Data collection and frame annotation**

**Table 4 Data diversity statistics table**

| Diversity | Classes | Number of frames | percentage |
| --- | --- | --- | --- |
| ray of light | Normal surgical scene lighting | 1108 | 60.0% |
|  | Dim lighting in a surgical setting | 487 | 26.4% |
|  | Strong surgical scene lighting | 252 | 13.6% |
| Blood infiltration | No infiltration | 814 | 44.1% |
|  | Partial infiltration（<50%） | 665 | 36.0% |
|  | Severe infiltration（>50%） | 368 | 19.9% |
| Organization and surgical instrument shielding | No shielding | 683 | 37.0% |
|  | Partial i shielding（<50%） | 624 | 33.8% |
|  | Severe shielding（>50%） | 540 | 29.2% |


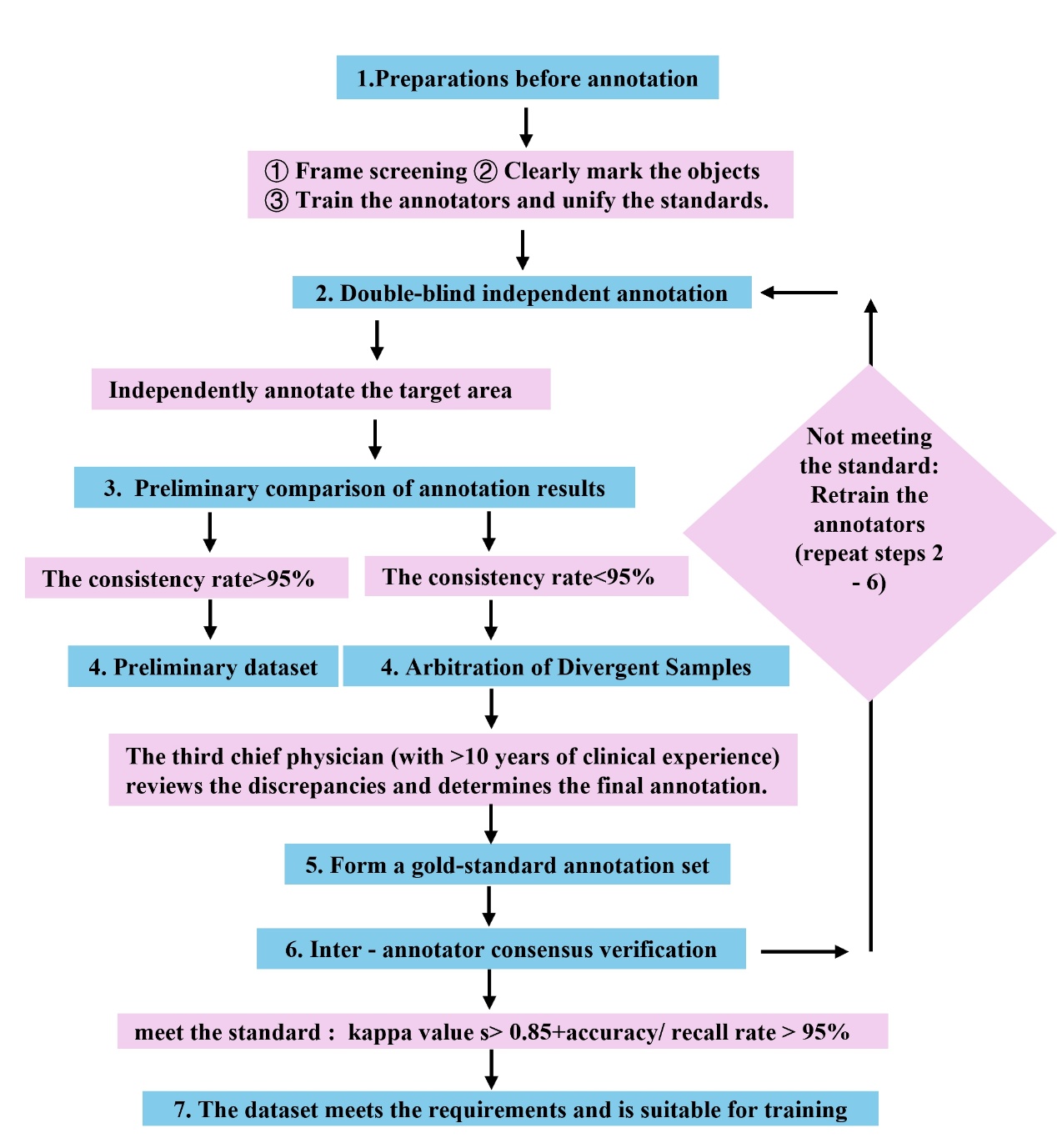


**Figure 2 Annotation flowchart**

**2.2 Experimental method**

**2.22 Model training, validation and testing**

**Table 5** **The Cross-validation experimental results of YOLOv8**

| C  -v | | P | | R | @0.5 | @0.5-0.95 | F1(%) | | P | R | @0.5 | | @0.5-0.95 | F1(%) | | |
| --- | --- | --- | --- | --- | --- | --- | --- | --- | --- | --- | --- | --- | --- | --- | --- | --- |
|  | | | Object detection | | | | | instance segmentation | | | | | | | |  |
| 1 | 0.953 | | | 0.881 | 0.950 | 0.637 | 91.6 | 0.979 | | 0.893 | | 0.955 | 0.649 | | 93.4 |  |
| 2 | 0.902 | | | 0.903 | 0.973 | 0.671 | 90.3 | 0.971 | | 0.913 | | 0.948 | 0.652 | | 94.1 |  |
| 3 | 0.956 | | | 0.887 | 0.943 | 0.667 | 92.0 | 0.962 | | 0.897 | | 0.956 | 0.638 | | 91.9 |  |
| 4 | 0.954 | | | 0.879 | 0.966 | 0.675 | 92.6 | 0.914 | | 0.905 | | 0.948 | 0.629 | | 91.0 |  |
| 5 | 0.914 | | | 0.865 | 0.976 | 0.697 | 87.3 | 0.966 | | 0.936 | | 0.979 | 0.688 | | 95.1 |  |
| 6 | 0.960 | | | 0.849 | 0.948 | 0.624 | 90.1 | 0.958 | | 0.921 | | 0.954 | 0.634 | | 93.9 |  |
| 7 | 0.952 | | | 0.878 | 0.950 | 0.671 | 91.4 | 0.971 | | 0.868 | | 0.948 | 0.592 | | 91.7 |  |
| 8 | 0.970 | | | 0.858 | 0.954 | 0.657 | 93.3 | 0.914 | | 0.889 | | 0.952 | 0.628 | | 90.1 |  |
| 9 | 0.960 | | | 0.867 | 0.949 | 0.641 | 91.1 | 0.979 | | 0.919 | | 0.973 | 0.632 | | 94.8 |  |
| 10 | 0.978 | | | 0.907 | 0.975 | 0.677 | 94.1 | 0.971 | | 0.894 | | 0.951 | 0.609 | | 93.1 |  |
| 11 | 0.933 | | | 0.926 | 0.946 | 0.667 | 93.0 | 0.945 | | 0.899 | | 0.949 | 0.635 | | 92.1 |  |
| 12 | 0.962 | | | 0.896 | 0.950 | 0.635 | 92.8 | 0.964 | | 0.907 | | 0.938 | 0.623 | | 93.5 |  |
| 13 | 0.952 | | | 0.912 | 0.929 | 0.672 | 93.2 | 0.967 | | 0.903 | | 0.968 | 0.647 | | 93.4 |  |
| 14 | 0.945 | | | 0.902 | 0.946 | 0.673 | 92.3 | 0.958 | | 0.924 | | 0.951 | 0.635 | | 94.1 |  |
| 15 | 0.989 | | | 0.891 | 0.951 | 0.694 | 93.7 | 0.966 | | 0.876 | | 0.972 | 0.633 | | 93.0 |  |
| 16 | 0.922 | | | 0.890 | 0.956 | 0.716 | 90.6 | 0.979 | | 0.851 | | 0.929 | 0.589 | | 91.1 |  |
| 17 | 0.958 | | | 0.889 | 0.901 | 0.673 | 92.2 | 0.963 | | 0.887 | | 0.959 | 0.678 | | 92.9 |  |
| 18 | 0.962 | | | 0.906 | 0.953 | 0.665 | 93.3 | 0.973 | | 0.915 | | 0.949 | 0.637 | | 94.3 |  |
| 19 | 0.978 | | | 0.859 | 0.904 | 0.681 | 92.2 | 0.956 | | 0.871 | | 0.958 | 0.646 | | 92.9 |  |
| 20 | 0.957 | | | 0.889 | 0.971 | 0.699 | 92.2 | 0.969 | | 0.931 | | 0.942 | 0.631 | | 95.0 |  |
| 21 | 0.962 | | | 0.928 | 0.952 | 0.672 | 94.5 | 0.971 | | 0.892 | | 0.957 | 0.599 | | 93.0 |  |
| 22 | 0.986 | | | 0.927 | 0.926 | 0.629 | 93.4 | 0.966 | | 0.908 | | 0.957 | 0.651 | | 94.1 |  |
| 23 | 0.974 | | | 0.905 | 0.940 | 0.677 | 93.8 | 0.974 | | 0.838 | | 0.955 | 0.635 | | 90.1 |  |
| 24 | 0.963 | | | 0.877 | 0.953 | 0.623 | 92.1 | 0.973 | | 0.881 | | 0.946 | 0.611 | | 91.6 |  |
| 25 | 0.964 | | | 0.926 | 0.956 | 0.703 | 94.5 | 0.938 | | 0.867 | | 0.977 | 0.676 | | 90.1 |  |
| 26 | 0.958 | | | 0.881 | 0.979 | 0.687 | 91.8 | 0.965 | | 0.892 | | 0.969 | 0.637 | | 92.7 |  |
| 27 | 0.983 | | | 0.939 | 0.975 | 0.676 | 96.1 | 0.979 | | 0.906 | | 0.917 | 0.664 | | 94.1 |  |
| 28 | 0.979 | | | 0.859 | 0.958 | 0.693 | 92.6 | 0.965 | | 0.848 | | 0.968 | 0.634 | | 90.3 |  |
| 29 | 0.942 | | | 0.851 | 0.923 | 0.672 | 89.4 | 0.923 | | 0.949 | | 0.951 | 0.593 | | 93.6 |  |
| 30 | 0.976 | | | 0.929 | 0.953 | 0.721 | 96.2 | 0.967 | | 0.906 | | 0.943 | 0.635 | | 93.6 |  |
| x̄: | 0.958 | | | 0.892 | 0.950 | 0.672 | 92.4 | 0.962 | | 0.896 | | 0.954 | 0.635 | | 92.8 |  |

Note：C-v：Cross-validation; P: Precision; R: Recall; x̄: Average; @0.5:Map@0.5；@0.5-0.95:Map@0.5-0.95.

**Table 6 The Cross-validation experimental results of YOLOv12**

| C-v | P | R | @0.5 | @0.5-0.95 | F1(%) | P | R | @0.5 | @0.5-0.95 | F1(%) |
| --- | --- | --- | --- | --- | --- | --- | --- | --- | --- | --- |
|  | Object detection | | | | | instance segmentation | | | | |
| 1 | 0.945 | 0.893 | 0.912 | 0.668 | 91.9 | 0.947 | 0.895 | 0.948 | 0.653 | 92.0 |
| 2 | 0.901 | 0.862 | 0.951 | 0.646 | 88.1 | 0.902 | 0.874 | 0.917 | 0.632 | 88.8 |
| 3 | 0.947 | 0.921 | 0.939 | 0.695 | 93.4 | 0.899 | 0.916 | 0.948 | 0.673 | 90.7 |
| 4 | 0.938 | 0.900 | 0.971 | 0.674 | 91.8 | 0.936 | 0.902 | 0.949 | 0.639 | 91.9 |
| 5 | 0.986 | 0.892 | 0.946 | 0.694 | 93.7 | 0.948 | 0.890 | 0.951 | 0.648 | 91.8 |
| 6 | 0.946 | 0.900 | 0.966 | 0.671 | 92.2 | 0.932 | 0.905 | 0.954 | 0.638 | 91.8 |
| 7 | 0.958 | 0.895 | 0.941 | 0.675 | 92.6 | 0.963 | 0.889 | 0.948 | 0.661 | 92.5 |
| 8 | 0.943 | 0.910 | 0.951 | 0.671 | 92.7 | 0.949 | 0.914 | 0.952 | 0.648 | 93.1 |
| 9 | 0.979 | 0.918 | 0.937 | 0.697 | 94.8 | 0.950 | 0.899 | 0.949 | 0.633 | 92.4 |
| 10 | 0.934 | 0.904 | 0.965 | 0.665 | 91.9 | 0.929 | 0.908 | 0.974 | 0.611 | 91.8 |
| 11 | 0.948 | 0.896 | 0.939 | 0.671 | 92.1 | 0.938 | 0.960 | 0.948 | 0.634 | 94.9 |
| 12 | 0.933 | 0.943 | 0.937 | 0.686 | 93.8 | 0.968 | 0.899 | 0.966 | 0.632 | 93.2 |
| 13 | 0.928 | 0.898 | 0.954 | 0.688 | 91.3 | 0.942 | 0.942 | 0.944 | 0.681 | 94.2 |
| 14 | 0.947 | 0.937 | 0.939 | 0.701 | 94.2 | 0.950 | 0.900 | 0.959 | 0.636 | 92.4 |
| 15 | 0.977 | 0.898 | 0.981 | 0.632 | 93.6 | 0.948 | 0.898 | 0.948 | 0.659 | 92.2 |
| 16 | 0.967 | 0.928 | 0.952 | 0.644 | 94.7 | 0.969 | 0.930 | 0.949 | 0.638 | 94.9 |
| 17 | 0.947 | 0.897 | 0.941 | 0.652 | 92.1 | 0.947 | 0.898 | 0.968 | 0.638 | 92.2 |
| 18 | 0.896 | 0.903 | 0.942 | 0.672 | 90.0 | 0.917 | 0.913 | 0.949 | 0.640 | 91.5 |
| 19 | 0.946 | 0.903 | 0.941 | 0.621 | 92.4 | 0.947 | 0.903 | 0.958 | 0.696 | 92.4 |
| 20 | 0.916 | 0.944 | 0.970 | 0.689 | 93.0 | 0.965 | 0.954 | 0.968 | 0.639 | 95.9 |
| 21 | 0.940 | 0.904 | 0.930 | 0.684 | 92.2 | 0.943 | 0.907 | 0.961 | 0.652 | 92.5 |
| 22 | 0.928 | 0.905 | 0.954 | 0.654 | 91.6 | 0.938 | 0.902 | 0.934 | 0.654 | 92.0 |
| 23 | 0.975 | 0.887 | 0.961 | 0.642 | 92.9 | 0.948 | 0.889 | 0.974 | 0.615 | 91.8 |
| 24 | 0.941 | 0.890 | 0.924 | 0.695 | 91.5 | 0.974 | 0.893 | 0.964 | 0.665 | 93.2 |
| 25 | 0.932 | 0.877 | 0.905 | 0.646 | 90.3 | 0.948 | 0.888 | 0.966 | 0.645 | 92.9 |
| 26 | 0.936 | 0.894 | 0.946 | 0.647 | 91.5 | 0.957 | 0.923 | 0.946 | 0.677 | 94.0 |
| 27 | 0.956 | 0.918 | 0.967 | 0.721 | 94.2 | 0.938 | 0.899 | 0.957 | 0.658 | 91.8 |
| 28 | 0.925 | 0.900 | 0.926 | 0.648 | 91.3 | 0.937 | 0.911 | 0.968 | 0.628 | 92.4 |
| 29 | 0.939 | 0.895 | 0.897 | 0.669 | 91.6 | 0.980 | 0.896 | 0.916 | 0.657 | 93.6 |
| 30 | 0.912 | 0.913 | 0.967 | 0.699 | 91.2 | 0.936 | 0.901 | 0.957 | 0.619 | 91.8 |
| x̄ | 0.942 | 0.904 | 0.945 | 0.671 | 92.3 | 0.945 | 0.907 | 0.953 | 0.647 | 92.6 |

Note：C-v：Cross-validation; P: Precision; R: Recall; x̄: Average; @0.5:Map@0.5；@0.5-0.95:Map@0.5-0.95；

**2.23** **Evaluation index**

**Table 7 Explanation of the observation indicators**

| Indicators | formulas | significance of surgical scenarios | Recommendation level |
| --- | --- | --- | --- |
| Precision | $\frac{\mathrm{TP}}{TP+FP}$ | High accuracy means that the auxiliary equipment can ensure that the surgeon accurately "cuts and removes" the tumor and lymph nodes, and "accurately tracks" the gauze. | Featured First |
| Recall | $\frac{\mathrm{TP}}{TP+FN}$ | This indicator is the most crucial in surgical scenarios. A low recall rate indicates a high rate of missed lymph node detections, which will lead to inaccurate postoperative staging and directly affect the subsequent treatment plan; a high recall rate means assisting the surgeon in quickly locating the gauze, avoiding the retention of the gauze in the abdominal cavity, and for the detection of tumor lesions, it means assisting the surgeon in quickly locating and improving the surgical efficiency. | Highly recommend |
| mAP@0.5 | Mean Average Precision(mAP)= $\frac{I}{n}\sum_{i=1}^{i=N} AP_{i}$  （intersection-over-union>0.5） | The core requirement of the surgical scenario is "rapidly locating key targets + avoiding fatal errors" (missed lymph node detection, leftover gauze, incorrect tumor localization). mAP@0.5 is a relatively loose metric, so the clinical significance of this indicator is slightly less. | Secondary recommendation |
| mAP@0.5-0.95 | Mean Average Precision(mAP)= $\frac{I}{n}\sum_{i=1}^{i=N} AP_{i}$  （intersection-over-union：0.5-0.95） | The core requirement of the surgical scene is the mAP@0.5-0.95 metric, which is a relatively strict indicator. It is a comprehensive and rigorous assessment of the model's ability to assist the surgeon. Therefore, the significance of this metric is superior to mAP@0.5. | Generally recommended |
| F1 | 2×$\frac{Precision \times Recall}{Precision+ Recall}$ | This indicator serves as a supplement to precision and recall rates, comprehensively evaluating from various perspectives the model's ability to assist surgeons in accurately reporting and avoiding missing the "correct reporting" and "no missing" of lymph nodes, tumors, and gauze in surgical scenarios. | Featured First |

Note: In the above formula, TP, FP, FN, and PN respectively represent true positive, false positive, false negative, and true negative.

**2.3Questionnaire**

**Questionnaire based on the assistance of artificial intelligence for investigators**

1 How old are you?

A.18-30 years old B.31-40 years old C.41-50 years old D.50-60 years old E. over 60 years old

2 What is your job title?

A.no title B. physician C. Attending physician D. Associate Chief Physician E. Chief Physician

3 Do you understand the concept of AI-assisted surgeons?

A.I have never heard of it at all. B. I've heard of it, but I'm not sure about its specific meaning. C.

Generally aware that artificial intelligence is involved in surgery-related work D. It is more clear

about its basic concepts and application scenarios. E. Very familiar, can describe its technical

principles in detail

4 The image shows a view under robot-assisted laparoscopy. Please identify the lymph nodes

in the picture and estimate how long it would take you.


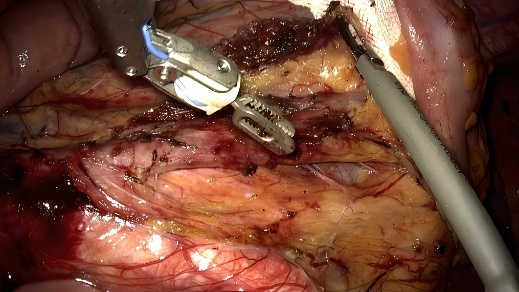


1. within two seconds B.2-10 seconds C.10-20 seconds D. more than 20 seconds E. Not found

5 This image shows the robot-assisted laparoscopic view after being labeled by artificial

intelligence. Using AI-assisted identification, identify the lymph nodes in the image and estimate


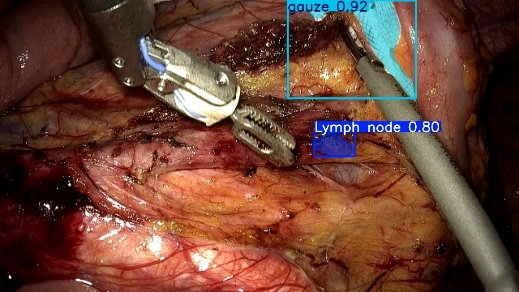


1. within two seconds B.2-10 seconds C.10-20 seconds D. more than 20 seconds E. Not found

6 In the previous question, if artificial intelligence is used in real-time to mark structures such

as 'lymph nodes' on the surgical screen during surgery, what is your view on the statement that

'artificial intelligence can improve surgical efficiency'?

A. I strongly agree B. very agreeable C. neutral (uncertain) D. Not very agreeable E. strongly

disagree

7 If you or a family member need surgery, would you prefer an AI-assisted surgical procedure

under similar conditions?

A. Very willing, believe in its advantages

B. Willing to try, but need to understand specific technical details.

C. It doesn't matter, follow the advice of the attending physician.

D. Not very willing, more trusting of purely manual surgery

E. completely unwilling

**3. Research Results**

**3.1 Data Annotation and Distribution**

**Table 8 Annotations made by two experts**

|  | Lymph Node | Gauze | Tumor |
| --- | --- | --- | --- |
| The number of instances where experts A and B agreed | 1620 | 635 | 970 |
| The number of instances that were marked by Expert A but not by Expert B | 1 | 0 | 2 |
| The number of instances that were marked by Expert B but not by Expert A | 1 | 2 | 1 |
| The number of instances that neither Expert A nor Expert B has labeled | 0 | 0 | 0 |

**Table 9 The situation noted by the two experts**

| Classes | The accuracy rate of annotations | The recall rate of annotations |
| --- | --- | --- |
| Lymph | 99.4% | 99.8% |
| Gauze | 99.6% | 100% |
| Tumor | 99.9% | 99.9% |
| All | 99.6% | 99.9% |


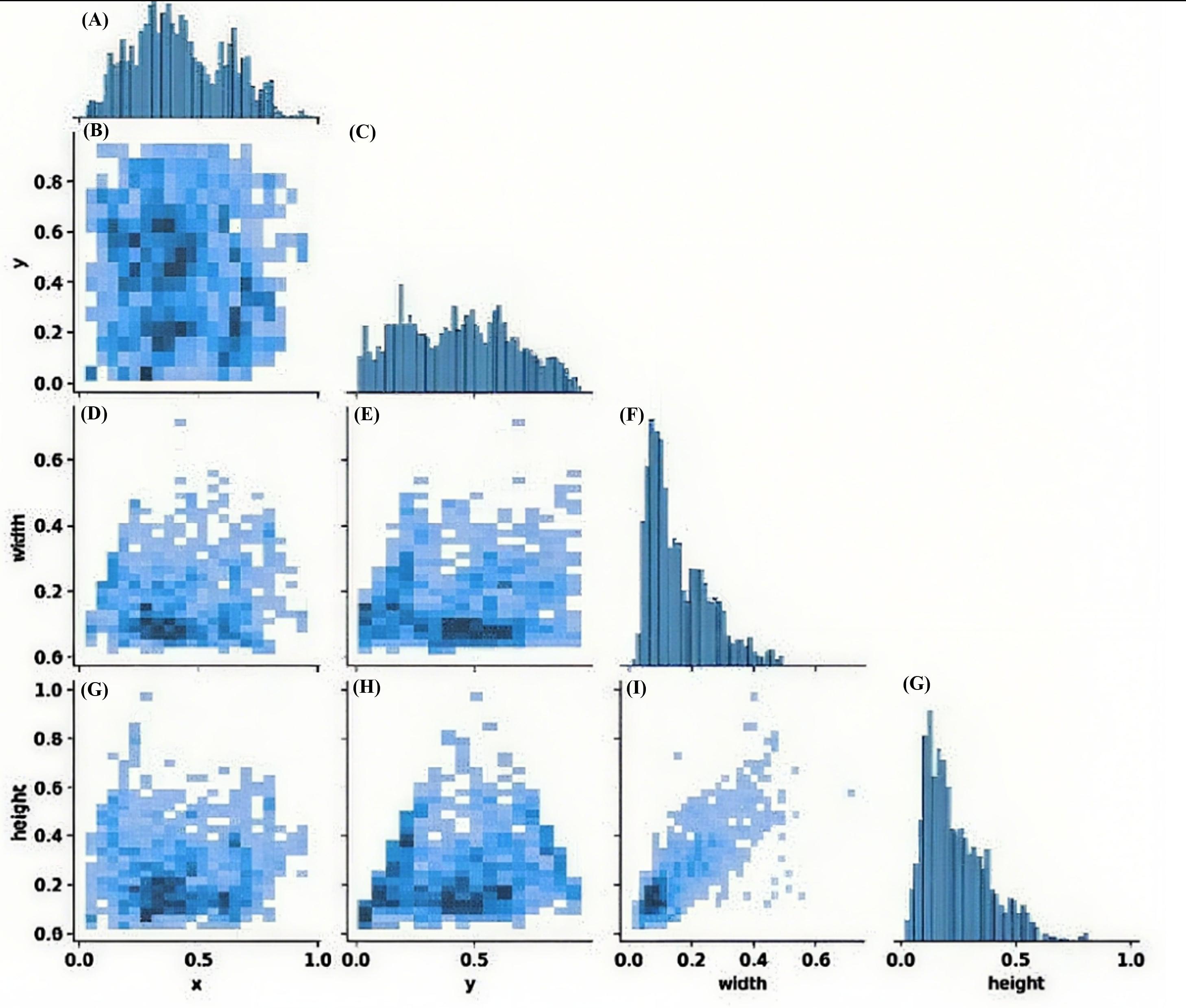


**Figure 3 Seaborn Pair Plot of Target Detection Training Dataset**

Note: (A): Histogram of variable x; (B): 2D histogram (heatmap) of x vs y; (C): Histogram of variable y;(D): 2D histogram of x vs width. (E): 2D histogram of y vs width; (F): Histogram of variable width; (G): 2D histogram of x vs height; (H): 2D histogram of y vs height.

(I): 2D histogram of width vs height;(G): Histogram of variable height.

**3.2 Model Training and Validation Results**


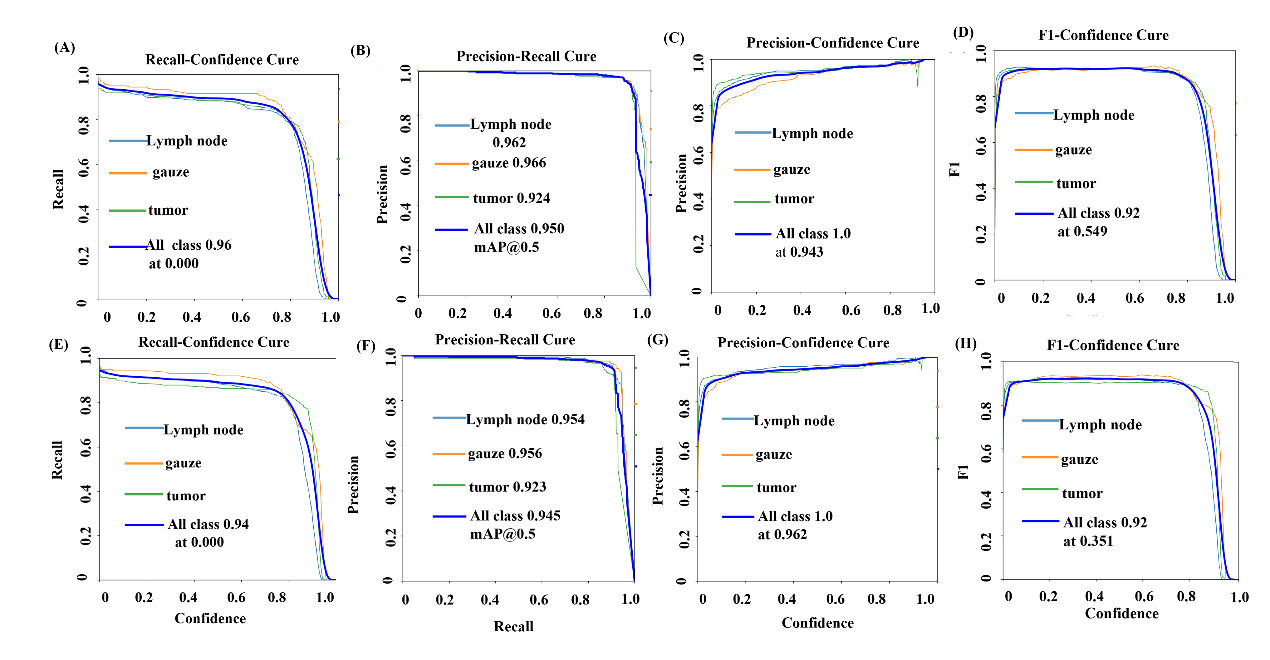


**Figure 4 Curve of target detection experimental results of YOLOv8 and YOLOv12**

**Note:** (A) and (E): Recall-Confidence Curve, Analyze the performance of the object detection model in terms of the confidence threshold for different classes in terms of the recall rate;

(B) and (F): Precision-Recall Curve, Analyze the dynamic relationship between "precision" and "recall" as reflected in object detection;

(C) and (G): Precision-Confidence Curve, Analyze the performance of the target detection model in terms of precision at different confidence thresholds;

(D) and H: F1-Confidence Curve, Analyze the F1 score performance of the target detection model under different confidence threshold values.

**3.3 Model Test Results**


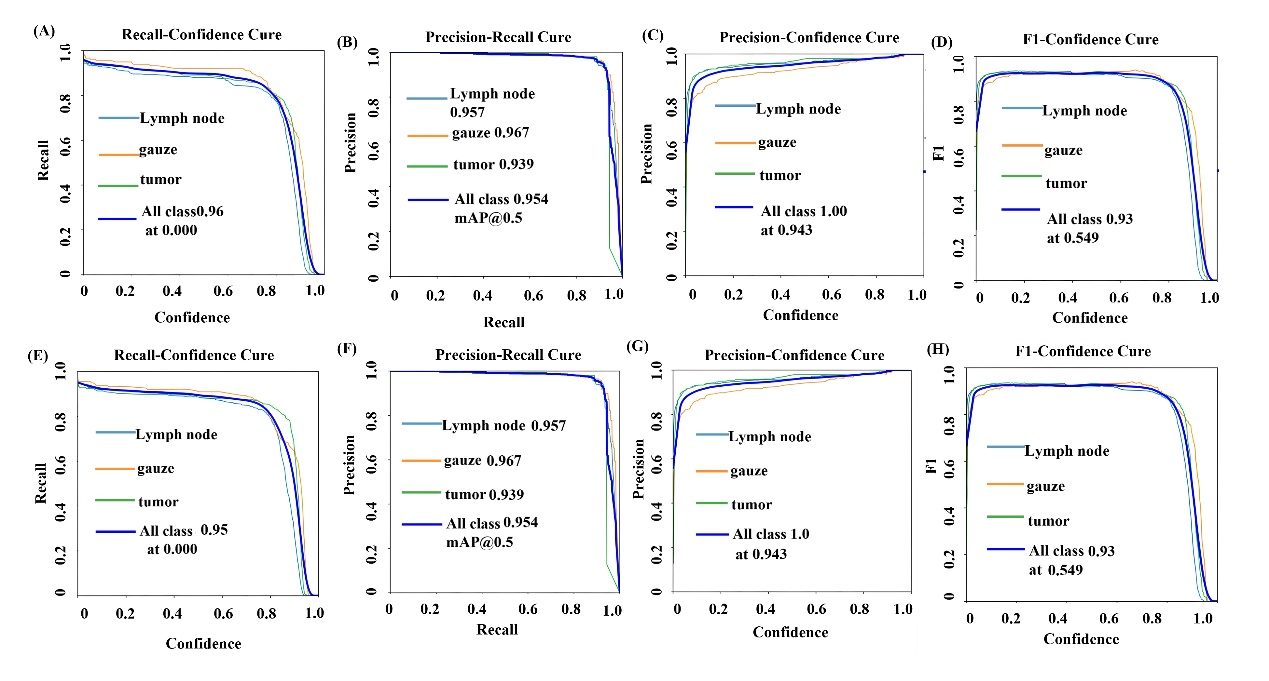


**Figure 5 Curve of instance segmentation experimental results of YOLOv8 and YOLOv12**

Note: (A)(B)(C) and(D)are the curve of experimental results of YOLOv8; (E)(F)(G) and(H)are the curve of experimental results of YOLOv12;(A) and (E:) Recall-Confidence Curve, Analyze the performance of the object detection model in terms of the confidence threshold for different classes in terms of the recall rate.

**Table 10 Comparison of statistic difference YOLOv8 and YOLOv12**

| Class | Precision | Recall rate | MAP@0.5 | MAP@0.5-0.95 | F1 score |
| --- | --- | --- | --- | --- | --- |
| Validation *  Testing *  Validation  testing | *P=*0.181  *P=*0.379  *P=*0.076  *P=*0.080 | *P=*0.049  *P=*0.037  *P=*0.046  *P=*0.031 | *P=*0.091  *P=*0.320  *P=*0.891  *P=*0.151 | *P=*0.673  *P=*0.385  *P=*0.270  *P=*0.179 | *P=*0.253  *P=*0.312  *P=*0.736  *P=*0.549 |

Note：The marked with "*" indicate target detection, while those without "*" indicate instance segmentation.

**Table 11 Comparison of statistic difference 0f YOLOv12 and** YOLOv12-seg

| Class | Precision | Recall rate | mAP@0.5 | mAP@0.5-0.95 | F1 score |
| --- | --- | --- | --- | --- | --- |
| Validation  Testing | *P*=0.171  *P*=0.385 | *P*=0.039  *P*=0.036 | *P*=0.043  *P*=0.045 | *P*=0.265  *P*=0.767 | *P*=0.589  *P*=0.476 |

**3.4 Training Efficiency and Adaptability**

**Table** **12 Training and validation efficiency of YOLOv8 and YOLOv12**

| Item | YOLOv8 | YOLOv12 |
| --- | --- | --- |
| The total duration of each training session | 98min | 135min |
| Frames per second | 370 FPS | 333 FPS |
| The size of the weight file | 6.8MB | 6.0MB |


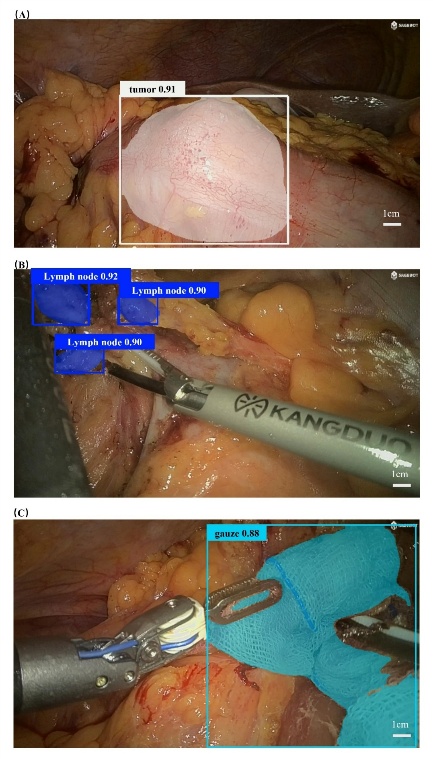


**Figure 6 The images extracted from the microscopic video frames using the YOLOv12 for inference**

Note: (A), (B), and (C) respectively represent the recognition results of tumors, lymph nodes, and gauze using YOLOv12.

- 1. **Questionnaire Survey Results**

**Table 13 Doctors of different ages failed to identify the condition of the lymph nodes**

| Age | AI assistance（%） | No AI assistance（%） |
| --- | --- | --- |
| 18-30 | 25.00% | 4.17% |
| 31-40 | 26.53% | 10.20% |
| 41-50 | 19.23% | 3.85% |
| 50-60  >60 | 0.00%  0.00% | 0.00%  0.00% |

Note: A：The lymph node was not identified in the absence of AI assistance; B: The lymph node was not identified in the presence of AI assistance

**Table 14 Doctors of different ages failed to identify the Condition of the lymph nodes.**

| Age | A | B |
| --- | --- | --- |
| 18-50 (Junior doctors) | 75 | 24 |
| >50 (senior doctors) | 1 | 0 |

Note：A: The lymph node was not identified in the absence of AI assistance（number）;B: The lymph node was not identified in the presence of AI assistance（number）

**Table 15 Comparison of lymph node identification time with vs. without AI assistance**

| Time | A | A(%) | B | B(%) | Standardized residual of A | Standardized residual of B |
| --- | --- | --- | --- | --- | --- | --- |
| Within two seconds | 1 | 93.00 | 32 | 29.91 | -5.27 | 5.27 |
| 2-10 seconds | 64 | 60.75 | 46 | 42.99 | 2.11 | -2.11 |
| 10-20 seconds | 12 | 11.21 | 14 | 13.08 | -0.50 | 0.50 |
| More than 20 seconds | 15 | 14.02 | 6 | 5.61 | 1.56 | -1.56 |
| Not found | 14 | 13.08 | 9 | 8.41 | 0.92 | -0.92 |

Note：A：The lymph node was not identified in the absence of AI assistance (number); B: The lymph node was not identified in the presence of AI assistance (number).


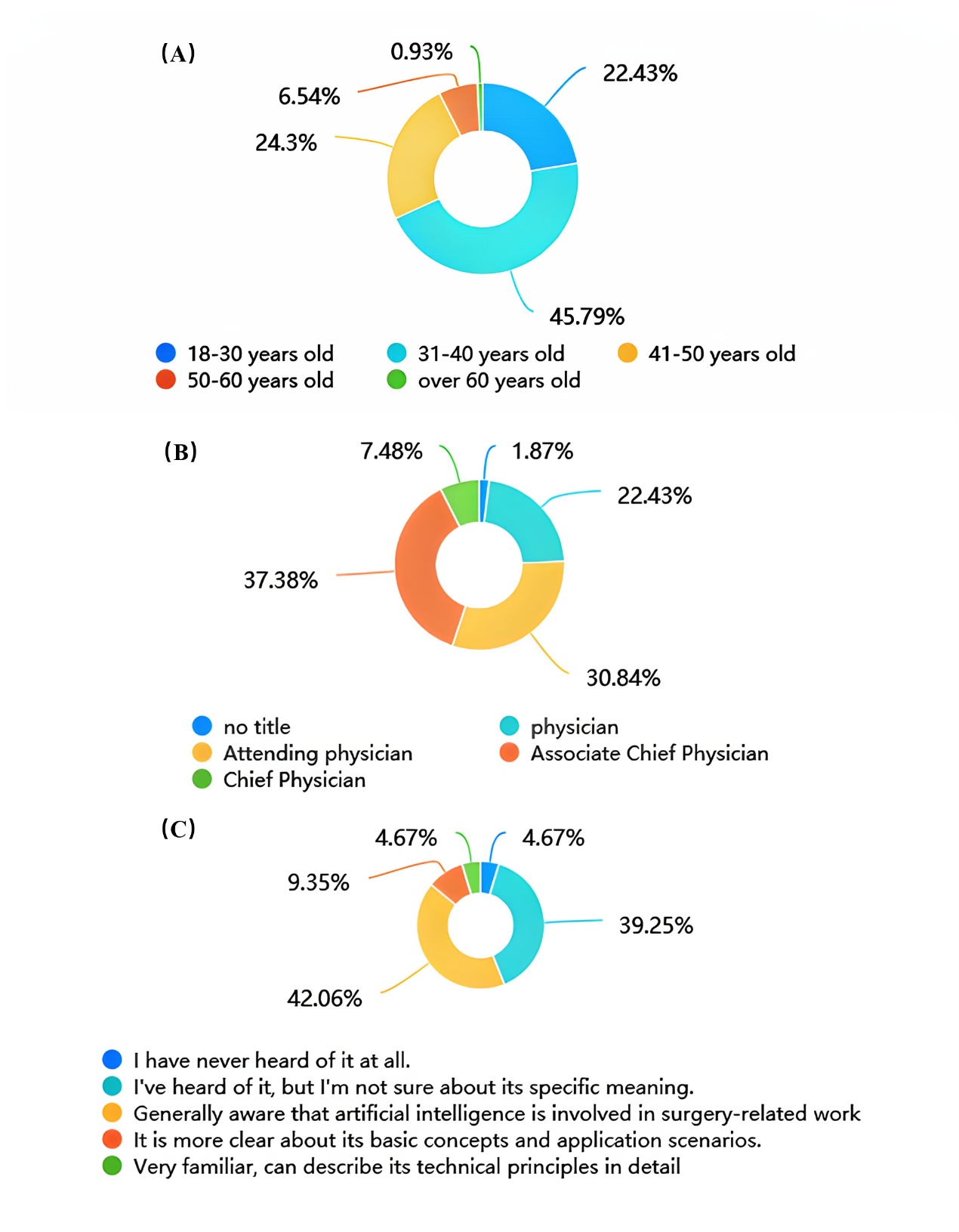


**Figure 7 The basic information of the general surgeon who participated in the questionnaire**

Note: (A): Age distribution chart of the participants in the questionnaire

（B）: Distribution chart of the job titles of the participants in the questionnaire

（C）: The level of understanding among the participants regarding the concept of "artificial intelligence-assisted surgeons"


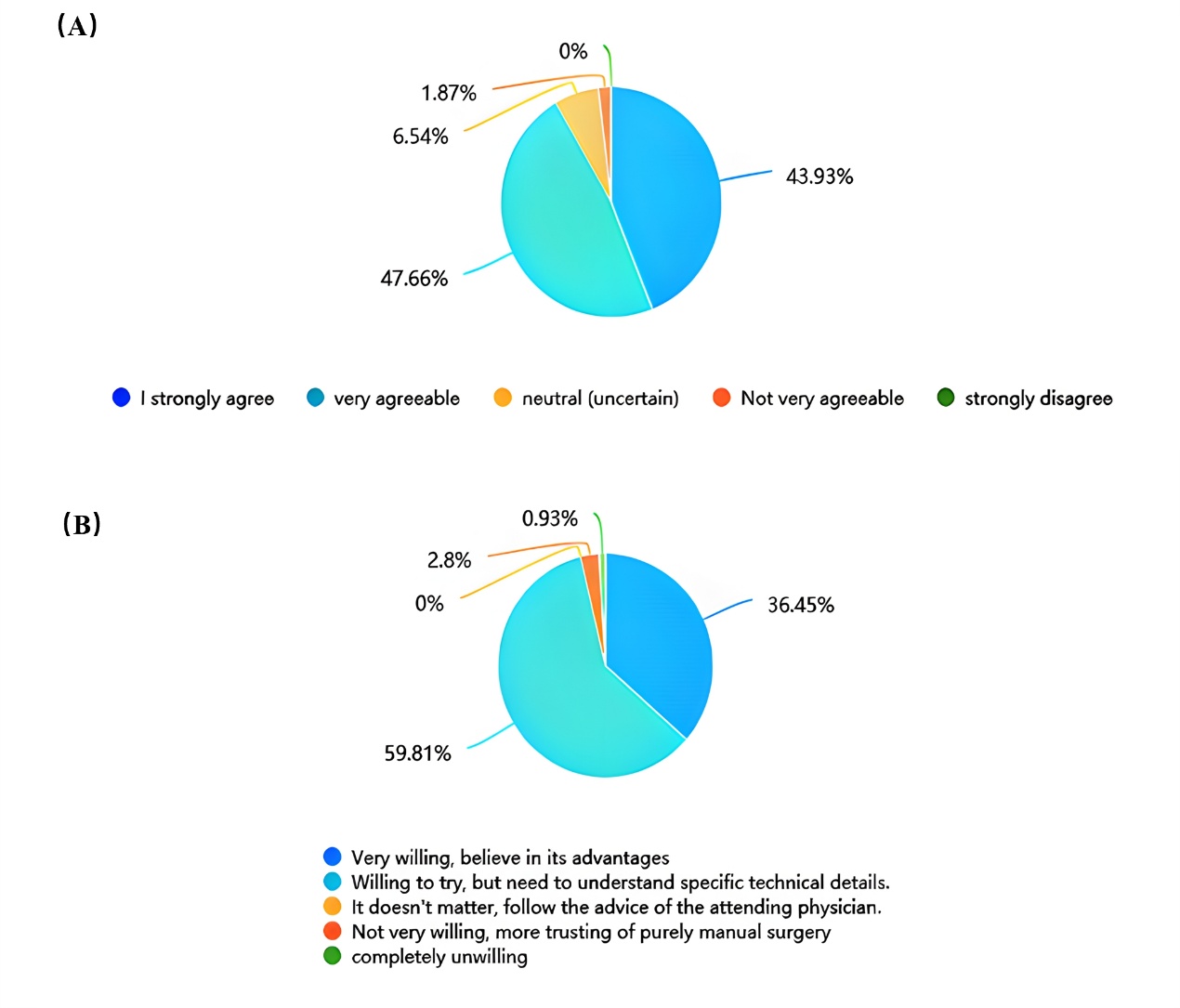


**Figure 8 The level of recognition that participants have for artificial intelligence technology**

**Note:** (A): The participants in the questionnaire completed the two questions above to compare the attitudes towards artificial intelligence technology before and after its use; (B): If you are a patient or a family member of a patient, under the same circumstances, your attitude towards the use of artificial intelligence technology to assist the surgeon would be.

**Total： （15 tables and 8 pictures）**
